# Identification and Characterization of a Novel Palmitoyl Acyltransferase as a Druggable Rheostat of Dynamic Palmitoylome in *L. donovani*

**DOI:** 10.3389/fcimb.2018.00186

**Published:** 2018-06-20

**Authors:** R. Ayana, Preeti Yadav, Rajesh Kumari, Dandugudumula Ramu, Swati Garg, Soumya Pati, Shailja Singh

**Affiliations:** ^1^Department of Life Sciences, School of Natural Sciences, Shiv Nadar University, Greater Noida, India; ^2^Special Centre for Molecular Medicine, Jawaharlal Nehru University, New Delhi, India

**Keywords:** palmitoylome, *Leishmania donovani*, motility, LdPAT4, chemical proteomics, 2-BMP

## Abstract

Palmitoylation has been recently identified as an important post-translational rheostat for controlling protein function in eukaryotes. However, the molecular machinery underlying palmitoylation remains unclear in the neglected tropical parasite, *Leishmania donovani*. Herein, we have identified a catalog of 20 novel palmitoyl acyltransferases (PATs) and characterized the promastigote-specific PAT (LdPAT4) containing the canonical Asp-His-His-Cys (DHHC) domain. Immunofluorescence analysis using in-house generated LdPAT4-specific antibody demonstrated distinct expression of LdPAT4 in the flagellar pocket of promastigotes. Using metabolic labeling-coupled click chemistry method, the functionality of this recombinant enzyme could be authenticated in *E. coli* strain expressing LdPAT4-DHHC domain. This was evident by the cellular uptake of palmitic acid analogs, which could be successfully inhibited by 2-BMP, a PAT-specific inhibitor. Using CSS-Palm based *in-silico* proteomic analysis, we could predict up to 23 palmitoylated sites per protein in the promastigotes, and further identify distinctive palmitoylated protein clusters involved in microtubule assembly, flagella motility and vesicular trafficking. To highlight, proteins such as Flagellar Member proteins (FLAM1, FLAM5), Intraflagellar Transport proteins (IFT88), and flagellar motor assembly proteins including the Dynein family were found to be enriched. Furthermore, analysis of global palmitoylation in promastigotes using Acyl-biotin exchange purification identified a set of S-palmitoylated proteins overlapping with the *in-silico* proteomics data. The attenuation of palmitoylation using 2-BMP demonstrated several phenotypic alterations in the promastigotes including distorted morphology, reduced motility (flagellar loss or slow flagellar beating), and inefficient invasion of promastigotes to host macrophages. These analyses confirm the essential role of palmitoylation in promastigotes. In summary, the findings suggest that LdPAT4 acts as a functional acyltransferase that can regulate palmitoylation of proteins involved in parasite motility and invasion, thus, can serve as a potential target for designing chemotherapeutics in Visceral Leishmaniasis.

## Introduction

Post-translational modifications (PTMs) constitute key mechanisms to increase the proteomic diversity and multi-functional attributes in a eukaryotic cell (Florens et al., 2002; Wastling et al., 2012). PTMs are catalyzed by enzymes and involve attachment of distinct amino acid chains, chemical moieties or peptide linkages. Palmitoylation, an essential but unexplored lipid-based PTM is controlled by Palmitoyl acyltransferases (PATs). This involves attachment of 16-carbon saturated fatty acid groups to specific cysteine residues in protein substrates through thioester linkages. This process acts as a biological rheostat for various processes in the eukaryotic system including cell signaling, neuronal transmission, cell motility, membrane trafficking, etc. (Fra et al., 2017). Emerging reports on trypanosomatids have also shown that myristoylation and palmitoylation might play roles in intracellular trafficking and parasite motility, however paucity exists in the knowledge regarding the role of palmitoylation in *Leishmania donovani* (Goldston et al., 2014).

Although computational identification of palmitoylation machinery has been established as a reliable and robust tool, it has not yet been utilized to identify the global palmitoylome and the respective PATs in *L. donovani*. For predicting palmitoylation sites, the existing algorithms like CSS-Palm rely on a clustering and scoring (CSS)-based strategy which classify experimentally verified palmitoylated sites from previous literature into Type I (sites follow a –CC– pattern, C is a cysteine residue), Type II (sites follow a –CXXC– pattern, C is a cysteine residue and X is a random residue) and Type III (random pattern site) clusters. As compared to other contemporary algorithms, the prediction performance of CSS-Palm is highly encouraging (Ren et al., 2008).

Previous studies from model organisms including higher mammals, *Drosophila* and yeast have demonstrated that PATs harbor a conserved Cysteine-rich domain (Asp-His-His-Cys, DHHC). PATs have been identified in several eukaryotic genomes, with 7 annotated genes in the *S. cerevisiae* genome and 23 in the human genome (Mitchell et al., 2006). Within the Apicomplexan family, *P. falciparum* and *T. gondii* each contain 12 and 18 PATs, whereas Trypanosomatid parasites like *T. brucei* encode 12 PATs. Additionally, comprehensive studies on PATs in other organisms have concluded that enzymes like DHHC2 and DHHC20 in *P. falciparum* and *H. sapiens* are essential for the respective cell survival, while the other PATs were found to be non-essential (Draper and Smith, 2010; Santos et al., 2015). Earlier reports on global palmitoylation in the apicomplexan parasites like *Plasmodium falciparum* have revealed that palmitoylation plays an essential role in schizont development and invasion (Jones et al., 2012). Concurrent pieces of evidence by Foe et al. suggest that palmitoylation also plays important roles in the formation and function of the glideosome complex in *Toxoplasma gondii* (Foe et al., 2015). More recently, over 400 palmitoylated proteins were identified in multiple cellular compartments, with a wide range of functions including metabolic processes, gliding, host-cell invasion as well as regulation of transcription and translation in *T. gondii* (Caballero et al., 2016). However, the role of palmitoylation has not been well established so far in case of *L. donovani*, the causative agent of deadly visceral leishmaniasis.

Although previous articles have discussed probable roles of palmitoylation in trypanosomatids, studies on PATs, PAT-mediated molecular mechanisms and their effect on the life cycle progression of *L. donovani* is still at their infancy (Goldston et al., 2014; Leroux and Krauth-Siegel, 2016). To address this, we have used an integrative biology approach which comprised of: (i) prediction of global palmitoylome and annotation of novel PATs using *in-silico* tools, (ii) *in-vitro* validation of global palmitoylation using clickable chemistry, (iii) cloning, expression and characterization of functional activity of the promastigote-specific LdPAT4 and (iv) validation of role of palmitoylation in motility and invasion of *L. donovani*. The overall *in-silico* approach used in this study has been compiled (Supplementary Figure 1). The findings have revealed a list of 20 previously unannotated PATs and their putative target clusters were found to be specifically associated with flagellar motility and invasion. Experimental study of global palmitoylation accentuated its role in parasite morphology, motility and invasion. We have further characterized the localization, expression and activity of a promastigote-specific PAT (LdPAT4-DHHC) and established its crucial role in parasite biology. To authenticate the catalytic activity of the recombinant LdPAT4-DHHC, we have expressed the same in a PTM-null model (*E. coli*). Finally, a chemical probe-based metabolic labeling method was utilized to establish the function of LdPAT4-DHHC. Overall, our study highlights global palmitoylation and its impact on the life cycle progression of *L. donovani* thus, suggesting LdPAT4 as a potential drug target for future pharmacotherapeutics.

## Materials and methods

### Computational prediction of protein palmitoylome

We used the stage-specific proteomics dataset by Nirujogi et al. (2014) as deposited in TriTrypDB (Aslett et al., 2010; Nirujogi et al., 2014). The promastigote and amastigote stage-specific proteins were delineated and the palmitoylome in each of the stages was characterized by CSS-Palm 4.0 (Ren et al., 2008). This stand-alone software is based on a CSS algorithm or 4th generation Group-based Prediction System, which is implemented on Java SE 6 for higher speed. We further filtered proteins having the highest number of palmitoylated sites from both promastigote and amastigote stages using a *medium* threshold, which correctly predicts five sites as a positive score in individual proteins. Using in-house scripts and *in-silico* analysis, we integrated the palmitoylated protein pool and the stage-specific protein expression data for identification of novel molecular targets.

### Target fishing and PPI network analysis

The pool of palmitoylated proteins was analyzed for significant Gene Ontology (GO) terms and critical modules were ascertained according to their molecular functions/associated pathways. The palmitoylated sites were predicted according to the aforementioned clustering types (I, II, and III) and the frequency of palmitoylation was assessed in all 1,225 promastigote-specific proteins. All the expression plots were made using RStudio (Team, 2015).

### Data mining and annotation of LdPATs

Data exploration was done using different parasite-specific databases including Plasmodium genomics resource database (PlasmoDB) (Aurrecoechea et al., 2009), Kinetoplastid genomics resource database (TriTrypDB) and Uniprot database for human-specific PAT sequences. Using TriTrypDB, putative PAT gene sequences in *L. donovani* were annotated based on conserved domain architecture and labeled (ID generation) in accordance with already annotated *L. infantum* sequences (Table 1). The 20 identified sequences were analyzed for the presence of functional and transmembrane domains. While, the domain architecture was assessed via SMART-Batch and INTERPRO online servers (Letunic et al., 2004; Mitchell et al., 2015), prediction of transmembrane and signal sequences was done using the Phobius server (Kall et al., 2007). We performed Multiple Sequence alignment (MSA) using MUSCLE algorithm (implemented in JALVIEW software) (Waterhouse et al., 2009). These sequences along with PAT sequences from three other organisms namely, *Homo sapiens, Plasmodium falciparum*, and *Trypanosoma brucei* were further used to construct a Neighbour-Joining (NJ) tree. The bootstrap consensus tree inferred from 600 replicates finally represented the evolutionary history of the taxa analyzed using MEGA6 software (Tamura et al., 2013). The evolutionary distances were computed using the Dayhoff matrix based method and are in the units of the number of amino acid substitutions per site. For an in-depth understanding of the intra-species relationships, we established a RAxml algorithm based tree using the phylogenetics-based ETE3 toolkit (Huerta-Cepas et al., 2016). A bubble-tree map was constructed to render clarity on the tree branches and their evolutionary patterns.

**Table 1:** *L. donovani* PAT gene annotation information in accordance with *Leishmania infantum* genomic sequences.

**TritrypDB gene ID**	**Putative naming**
LdBPK_030460.1	Hypothetical protein, conserved (Hp1)
LdBPK_040510.1	PAT1
LdBPK_090690.1	PAT5
LdBPK_111080.1	PAT7a
LdBPK_111090.1	PAT7b
LdBPK_130540.1	PAT6
LdBPK_140070.1	Hypothetical protein, conserved (Hp2)
LdBPK_211640.1	PAT3
LdBPK_221200.1	PAT11
LdBPK_231690.1	PAT12
LdBPK_231700.1	DHHC zinc finger domain-life protein (ZnFprot2)
LdBPK_231710.1	Hypothetical protein, conserved (Hp3)
LdBPK_242360.1	Zinc-finger multi-pass transmembrane protein (ZnFprot1)
LdBPK_252390.1	PAT10
LdBPK_280220.1	PAT9
LdBPK_282010.1	PAT4
LdBPK_301230.1	Hypothetical protein, conserved (Hp4)
LdBPK_330240.1	PAT2
LdBPK_344130.1	Hypothetical protein, conserved (Hp5)
LdBPK_352630.1	Hypothetical protein, conserved (Hp6)

### *In-vitro* cultures of *L. donovani* and macrophages

*Leishmania donovani* AG83 strain was cultured to conduct this study. Promastigotes were grown at 27°C in M199 media complemented with 10% heat-inactivated (30min, 56°C) fetal bovine serum (HIMEDIA) and 0.02 mg/mL gentamycin (Life Technologies, USA). Log phase promastigotes were used throughout the study. Parasite count of 10^6^ cells/ mL was maintained for all experiments unless specified otherwise. To study host macrophage invasion, we cultured the murine macrophage cell line (J774.A1) in complete RPMI media, supplemented with 10% FBS and 1X Pen Strep solution. The experiments were conducted using macrophage culture cells which did not exceed 10 passages. Healthy cells were counted and used for the invasion study.

### Growth inhibition assay

Cytotoxicity of 2-Bromopalmitate (2-BMP), a specific inhibitor of palmitoylation in *L. donovani* cells was evaluated by monitoring the release of Lactate Dehydrogenase (LDH) from promastigotes by using CytoTox 96 Non-Radioactive Cytotoxicity Assay kit (Promega, USA), a colorimetric assay for analysis of cell death. The assay was performed as per manufacturer's instructions. Briefly, parasites harvested in the exponential log phase were seeded in 96-well plate maintaining a constant well volume as 100 μL and triplicates for both control and test samples. Parasites were subjected to respective drug treatments for 72 h, and Amphotericin B (2.5 μg/mL-Sigma Aldrich) was considered as a positive control. Perceptual cytotoxicity of 2-BMP was calculated by normalizing with Amphotericin B as 100%. Similarly, cell proliferation assay was performed for testing the cell viability and determining the IC_50_ concentration of 2-BMP.

### Macrophage invasion assay

Invasion and proliferation efficiency of promastigotes in the human macrophages was evaluated in the presence and absence of drug. Briefly, macrophage cells were cultured and seeded onto a six-well plate at a maintained cell density of 5 × 10^5^ as discussed previously. For infection, metacyclic promastigotes were added to the macrophages at a ratio of 10:1. Control (Untreated), positive control (Amphotericin B) and stained control (Uninfected Macrophages) were maintained for this experiment. After 6 h, uninfected promastigotes were removed, and fresh media was added to the cells followed by incubation for 72 h. Infected macrophages were treated with 2-BMP at its IC_50_ concentration and incubated for 72 h. The invasion efficiency of control and treated samples was assessed by visualization and counting of intracellular parasite load using Giemsa (Sigma-Aldrich) staining.

### Evaluation of parasite motility

Motility of *L. donovani* promastigotes was observed using Time-lapse video microscopy. Briefly, promastigotes in their mid-log phase were treated with 2-BMP for 72 h, with untreated cells maintained as the control. Following treatment, parasites were washed twice with PBS and further re-suspended in 50 μl of PBS. Samples were visualized under the NIKON Ti Eclipse microscope for real-time monitoring of parasite flagellar motility.

### Flagella beat parameters

For the analysis of flagella beating movement, videos of actively beating flagella of individual parasite cells were captured. Following this, the wavelength for the beats of individual cells were determined by using the “2 points” tool under “Measure” tab for measuring the length between the minima and maxima of processed images of flagella. The image processing analysis was performed using the Nikon NIS Analysis software 4.54, 64 bit. The flagella speed was calculated as the dividend of the total wavelength covered by flagella during the total time lapsed (60 s).

### Click chemistry

Palmitoylation was examined by labeling with Click Tag™ Palmitic Acid Alkyne, a 16-carbon saturated fatty acid group combined with a specific azide dye (Oregon Green® 488). Briefly, *L. donovani* promastigotes at their log phase were cultured at 26°C for 24 h and treated with 2-BMP (Sigma). Palmitic acid (Alk-C16, Cayman Chemical) was dissolved in DMSO to achieve a final stock concentration of 50mM. Drug control was maintained without any drug addition, whereas the promastigote sample without drug and Alk-C16 was used as unstained control. After drug treatment, parasites were incubated overnight with 100μM Alk-C16 (except labeling control) at 26°C. Parasites were washed twice with cold PBS and fixed with chilled methanol for 5min, and further permeabilized using 0.1% Triton X-100 (Sigma) in PBS at RT for 5min. These processed parasite samples were subjected to a click labeling reaction in 100 μl of dye mix containing 0.1mM azide dye (Oregon Green® 488, Thermo Fisher Scientific), 1 mM Tris (2-carboxyethyl) phosphine hydrochloride (TCEP-Sigma) and 1mM CuSO_4_ (Sigma) in water. Images were acquired by fluorescence microscope (NIKON Ti eclipse). Samples were also examined by flow cytometry (FACS-BD LSR Fortessa) and analyzed by FlowJo software.

### *Leishmania donovani* palmitoylome purification using acyl-biotin exchange method

Acyl-biotin exchange (ABE) purification of whole parasite lysate was carried out using the established method as published by Wan and colleagues, with the following modifications (Wan et al., 2007). Parasites were resuspended in 4ml of lysis buffer containing 10mM NEM. The rest of the procedure was performed as described. Silver staining was performed to analyse purified fractions.

### Mass-spectroscopy

Following ABE purification, the protein extracts were pooled from individual gel lanes and LC-MS was conducted. The resultant chromatograms were analyzed for heavily palmitoylated proteins, and specifically the 2-BMP treated samples were analyzed for probable alternation in palmitoylation pattern. The chromatograms were analyzed using Proteome Discoverer software (Thermo Fisher Scientific) and a cut-off of FDR or *q* < 0.5 was considered significant. The coverage, number of unique peptides, the critical Seq score and confidence of individual peptides were noted.

### Quantitative real-time PCR

Primers were designed with amplicon sizes of 100–200 bp for all 20 LdPAT sequences and other promastigote control genes using Primer3Plus software (Untergasser et al., 2007; Supplementary Table 1). Total RNA was isolated from healthy promastigote culture using Qiagen RNA Isolation kit. Purity of RNA was tested using NanoDrop and subsequently, cDNA was isolated and checked on 1% Agarose gel. The expression of 20 LdPATs was quantified using Real Time PCR (RT-PCR). Transcript-level expression of several annotated PAT-specific target genes like Dynein1, Dynein2, FLAM1, FLAM5, etc., housekeeping controls (40S, actin, ATPase) as well as promastigote-specific marker (LACK) were analyzed.

### *In-silico* structural studies

The sequences of 20 novel LdPATs were obtained from TriTrypDB and their structural models were constructed using the I-TASSER server (Zhang, 2008). The predicted theoretical models were visualized with PyMOL Molecular Graphics System, Version 1.7.4 (Delano, 2002). Using ModRefiner software, the structure was further refined for future docking studies (Xu and Zhang, 2011). Quality validation of the resultant models was done with RAMPAGE. We specifically examined the drug-protein interaction between 2-BMP inhibitor and LdPAT4. The 3D structure of 2-BMP was downloaded from PubChem database in SDF format and converted to standard PDB format. For all molecular visualization of the initial and docking models, PYMOL 1.7.4 and AutoDockTools were used (Morris et al., 2009). As per the predicted active sites by I-TASSER COACH server, we ensured the active site residues were covered when constructing the virtual pocket/grid for docking. Using these predicted residues, a virtual 3D grid of 32 × 36 × 28 Å measurements with default spacing and exhaustiveness = 8 was constructed through the Autogrid module of AutoDockTools (Morris et al., 2009). We performed molecular docking studies using Autodock Vina with compounds to rationalize its activity against LdPAT4 (Trott and Olson, 2010). The top-ranked conformations of compound within the protein catalytic pocket were selected based on the lowest free binding energies. The most stable conformations were visualized for polar contacts and hydrogen bonds.

### LdPAT4-specific cloning, expression, and antibody generation

Protein-level expression of 20 LdPATs in the promastigote and amastigote stages was evaluated using available *in-silico* proteomics data. Specifically, we focused on LdPAT4 as it was strictly expressed in the promastigote stage. The 423 bp DHHC domain of *L. donovani* PAT4 was amplified using specific primers: Fwd 5′-CTCGGATCCAGCTTGTGGGAGGATGT-3′, Rev 5′-GCAGTCGACTTAGTTGCGCTTGTGAGC-3′ and cloned into the pGEX-4T-1 vector (GE Healthcare) at Bam-HI and Sal-I sites and expressed in *E. coli* Rosetta cells. The resultant clone is labeled as LdPAT4-DHHC. Transformed Rosetta cells were then induced for expression of recombinant LdPAT4-DHHC tagged with GST using 1mM IPTG at 37°C for 4 h. The cell biomass was resuspended in the lysis buffer containing 50mM Tris-Cl, 100mM NaCl, 1mM DTT, 5% Glycerol, 1mM PMSF, 1X PIC, Lysozyme (10μg/ml) in PBS. Antibody was generated against LdPAT4-DHHC protein in Swiss albino mice. Each mouse was immunized by 3 subcutaneous injections (including first and second boost dose) of 0.25 mg 40 kDa antigen. At 0th day, prime dose was administered to mice. Subsequently, the first boost was given at 21st day and on the 35th day, first bleed was collected. The second boost was given on 42nd day and the final bleed was collected on the 56th day.

### Immunofluorescence staining

For the intracellular localization of LdPAT4-DHHC, promastigotes were immobilized on poly-L-lysine-coated coverslips. The cells were fixed and permeabilized followed by incubation with the anti-LdPAT4-DHHC antibody (1:1,000) for 1 h at RT. Subsequently, the cells were washed and then incubated for 45 min with the Alexa 546-conjugated Goat anti-rabbit (H+L) IgG antibody (Invitrogen). The nuclear and kinetoplastid DNA were stained with DAPI (Invitrogen). Immunofluorescence staining of parasites was visualized under confocal laser scanning microscope (Nikon).

### Immunoblotting

Lysate containing LdPAT4-DHHC was loaded onto 1ml Bioscale™ Mini Profinity™ GST cartridge (Biorad). Desired protein was eluted with 10 and 20mM reduced glutathione dissolved in 50mM Tris (pH 8.0). The eluted fractions were further checked for the presence of LdPAT4-DHHC using SDS-PAGE and Coomassie Brilliant Blue staining, and further confirmed by Western blotting using Anti-GST antibody.

### Evaluation of recombinant LdPAT4-DHHC activity

Cultured LdPAT4-DHHC/pGEX-4T-1/Rosetta cells were induced using 0.5mM IPTG along with addition of 100μM Alk-C16 for 6 h at 37°C. Following 6 h of induction, cells were fixed and permeabilized using ice-cold methanol and 0.01% TritonX-100 for 5 min at RT. Then, washing was done with 1X PBS followed by addition of 100μL dye mix containing 0.1mM azide dye (Oregon Green 488™, Thermo Fisher Scientific), 1mM Tris (2-carboxyethyl) phosphine hydrochloride (TCEP-Sigma) and 1mM CuSO_4_ (Sigma) dissolved in water. Finally, cells were mounted with DAPI anti-fade solution and observed under fluorescence microscope (Zeiss Apotome).

### Ethical statement

The animal protocol was ethically approved by Institutional Animal Ethics Committee (IAEC) of Jawaharlal Nehru University (JNU), New Delhi, India. Animal experiments were conducted at Central Laboratory Animal resources (CLAR), JNU.

### Statistical analysis

Student's *t*-test was performed to evaluate significant differences between treatment and control samples. *P* < 0.05 was significant and has been mentioned wherever required. Results represent the mean ± SD of minimum three independent experiments.

## Results

### LdPAT sequences are consistently conserved within and outside species

**Figure 1:**
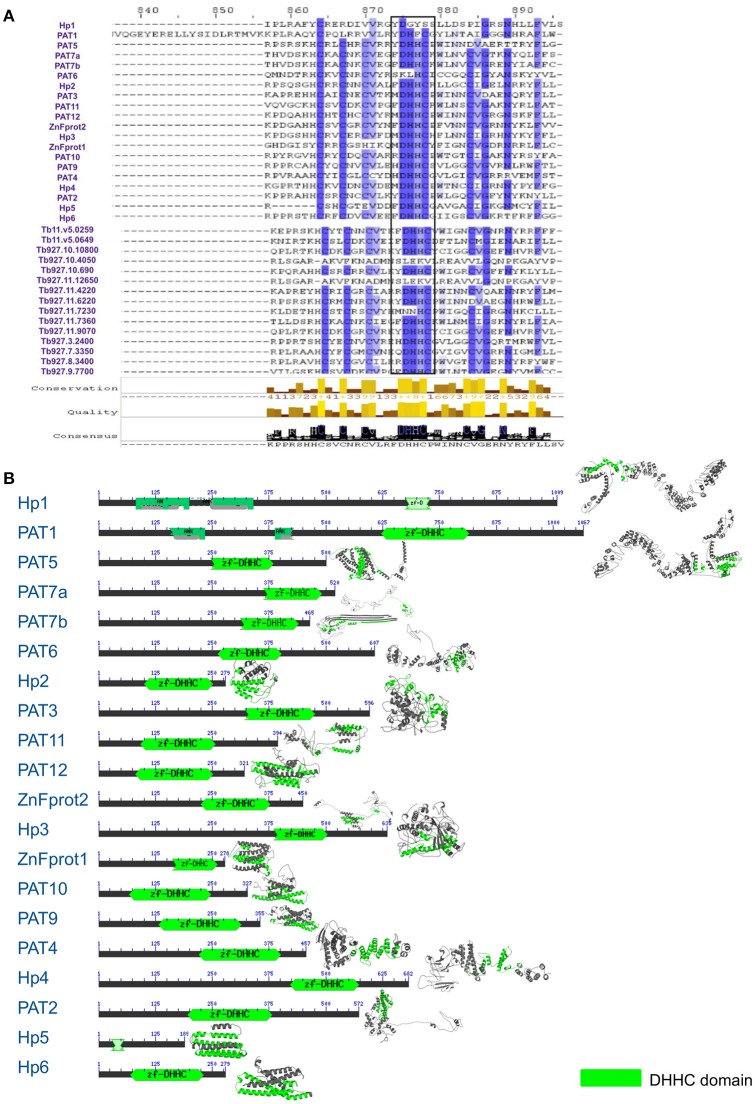
**(A)** Multiple sequence alignment showing conservation between *L. donovani* and *T. brucei* Palmitoyl acyltransferase sequences. **(B)** Domain architecture of 20 DHHC-containing gene sequences with annotation according to *L. infantum*.

Using various databases like the PlasmoDB, TriTrypDB and UniProt, we extracted PAT sequences of different organisms. Notably, none of the *L. donovani* PAT sequences were annotated and only minimal annotation information of related species was available. Using the annotated sequences of closest relative species *L. infantum*, we could annotate the unidentified 20 putative LdPAT protein sequences. Multiple Sequence Alignment analysis showed that the signature DHHC motif is highly conserved in all 20 PAT sequences, with similarity to the PAT DHHC domain of *T. brucei*, an extensively studied member of the Trypanosomatids (Figure 1A). Since the DHHC motif is a signature of the catalytic domain among PATs, we investigated the domain architecture which revealed interspecies motif conservation (Figure 1A). While numerous transmembrane regions were detected (2–6) without any signal peptide sequences, 2 of the members, PAT1 and hypothetical protein 1 were found to contain Ankyrin repeats (Figure 1B).

Validation of LdPATs and their probable downstream palmitoylated partners demonstrated consistent transcript-level expression in promastigotes, as observed in both semi-quantitative and quantitative PCR-based analyses (Supplementary Figure 2, Supplementary Tables 2, 3). The findings also showed the expression of several downstream effectors of LdPATs, such as motor assembly protein family (dynein1, dynein2, dynein4, dynein5), dynein assembly protein (ODA7) and flagellar member proteins (FLAM1, FLAM5), which strongly indicated the presence of healthy, motile promastigotes (Supplementary Figure 2).

To understand the phylogenetic relationships within this group of proteins, we evaluated their evolutionary proximity to other protozoan parasites and relationship to *Homo sapiens* by constructing an inter-species phylogenetic tree. To highlight, LdPATs showed significant closeness to TbPAT sequences with prominent clustering within same branches (Supplementary Figure 3A). Using ETE3 toolkit, we further analyzed the evolutionary relationships between the different species of *Leishmania* and found that most of the *L. donovani* sequences were clustering with high confidence (indicated by larger bubble size) with *L. infantum* and other species. The *Trypanosoma* outgroup species was seen to be the most distant from all sequences in the tree, thus validating the overall reliability of the tree. Notably, the inter-species tree has highlighted high conservation of specific PAT branches whereas, the intra-species tree represented a cluster of shortlisted PAT branches (LdPAT4 and LdPAT6) (Supplementary Figure 3B). However, the structural superimposition of all the 20 *L. donovani* PAT protein DHHC domains did not reflect high similarity (RMSD = 15.12). Differences in the secondary structure of 20 PATs also indicated the structural diversity amongst these proteins (Figure 1B).

### Characterization of novel LdPATs revealed LdPAT4-DHHC as a distinct marker of the promastigote stage

**Figure 2:**
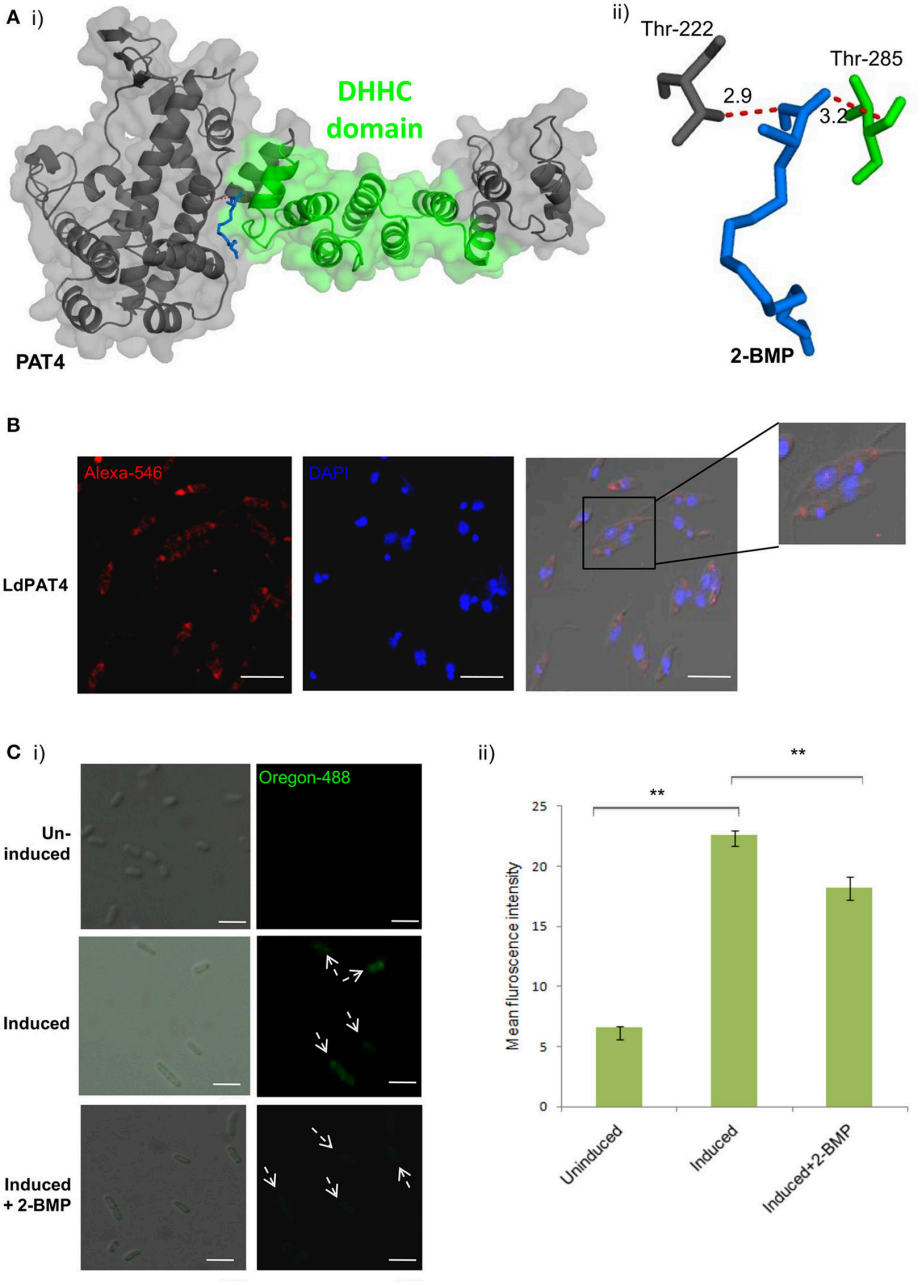
**(A)** Docking study of LdPAT4 with palmitoylation inhibitor 2-BMP. **(i)** The protein accessible surface area was visualized for flexible ligand docking. **(ii)** Strong H-bonding indicated key residues involved in the interaction of the catalytic domain of LdPAT4 to 2-BMP. **(B)** Immunofluorescence analysis demonstrating expression and cellular localization of LdPAT4 in *Leishmania* promastigotes. Scale bar in white indicates 5 μM. **(C) (i)** Normaski and fluorescence images showing the catalytic activity of LdPAT4-DHHC using click chemistry in transformed *E. coli* cells. Scale bar in white indicates 2 μM. **(ii)** Fluorometer readings showing uninduced, induced and 2-BMP treated LdPAT4-DHHC expressing *E. coli* cells (**p < < 0.05). Data are represented as mean ± SEM.

Out of the 20 annotated PATs, we characterized LdPAT4, a canonical PAT specific to the infective stage of the parasite (promastigote). The drug-protein interactions between 2-BMP and LdPAT4 protein were assessed computationally. We constructed a structural homology model of LdPAT4 and this structure was validated using RAMPAGE, and most of the residues were restricted to the favored region (90%) of the Ramachandran plot. The binding affinity of 2-BMP to the catalytic pocket was found to be−5.1 Kcal/mol. The bound protein-ligand complex revealed two strong hydrogen bonds among Thr222 and Thr285 of the catalytic pocket and O atoms of 2-BMP having 2.9 and 3.2 Å bond lengths respectively (Figures 2Ai,ii).

To further evaluate the expression and localization of LdPAT4-DHHC, Rosetta cells were transformed with pGEX-4T-1 expressing the recombinant form of LdPAT4. The recombinant protein could be detected in elute fraction three of transformed Rosetta cells (Supplementary Figure 4A). Western blotting using the in-house generated specific antibody validated the expression of LdPAT4-DHHC (Supplementary Figure 4B). Immunofluorescence analysis further revealed distinct expression of LdPAT4-DHHC in the flagellar pocket of *L. donovani* promastigotes (Figure 2B). Since the flagellar pocket exhibits crucial roles in motility as well as endocytotic processes, localization of the same protein to the pocket indicates a prominent role of this palmitoyl acyltransferase (LdPAT4) in the motile stage of *L. donovani*. Furthermore, to confirm the enzymatic activity of the recombinant LdPAT4-DHHC, we performed click chemistry method in a PTM-null prokaryotic system (*E. coli*). Upon expression of LdPAT4-DHHC in transformed *E. coli* cells, we found a substantial increase in fluorescence indicating lucid palmitoylation in these cells, whereas no fluorescence was detected in the uninduced *E. coli* cells (Figures 2Ci,ii). Conversely, following 2-BMP treatment of induced cells, reduction in fluorescence intensity was detected which was confirmed by fluorometric analysis (Figure 2Cii).

### Palmitoylome profiling identified distinct protein clusters in promastigote vs. amastigote stages

**Figure 3:**
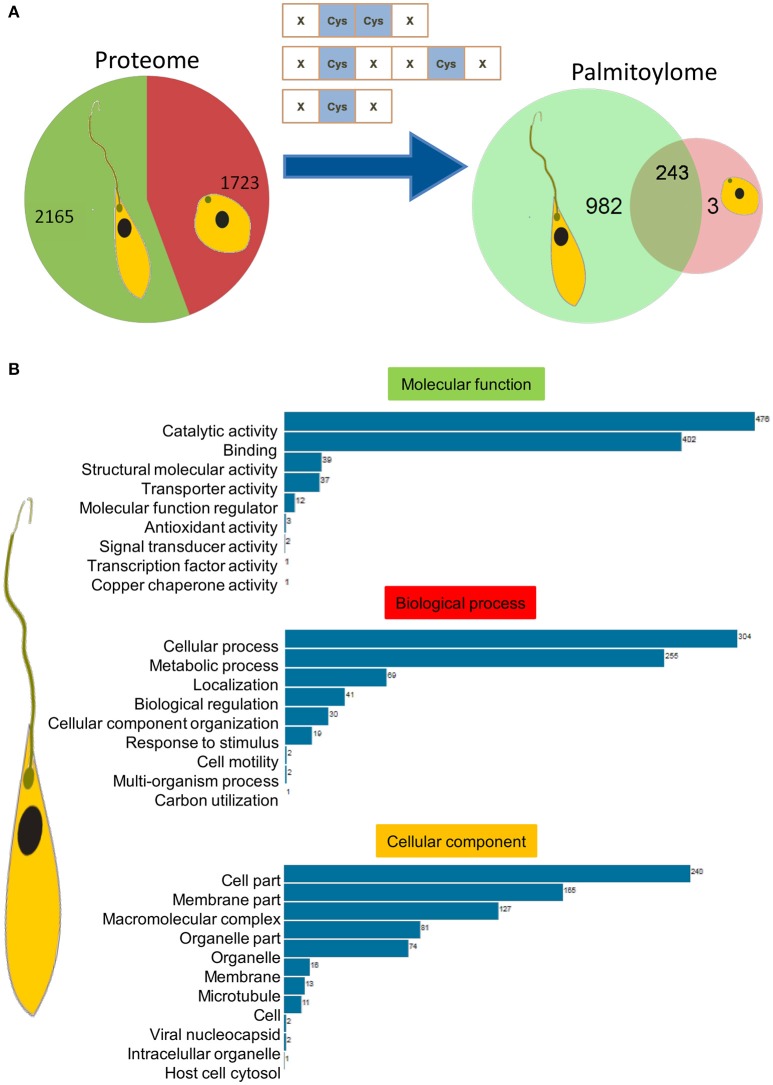
**(A)** Pie charts represent expressed proteins in motile vs. non-motile parasites, with respective palmitoylome statuses. **(B)** GO analysis of significantly palmitoylated (1225) proteins enriched critical biological processes relevant to parasite biology.

Parasite stage-specific proteomics demonstrated differential expression of 2,165 and 1,723 proteins belonging to promastigote and amastigote stages respectively (Figure 3A). Using the CSS-Palm prediction software, we found a confident set of proteins with evident palmitoylation and the rate of palmitoylation represented at least one palmitoylation site in each protein (if any). Based on the analysis, we could predict 1,225 and 246 palmitoylated proteins in the promastigote and amastigote protein pools respectively. This indicates significantly enhanced palmitoylation (~60%) in the promastigotes in comparison to amastigotes (Figure 3A). Interestingly out of 246 palmitoylated proteins in the amastigotes, only 3 were found to be unique to the stage (Figure 3A). The *in-silico* proteomic analysis of *L. donovani* stages also demonstrated significant expression of two PATs (LdPAT6 and LdPAT4) in promastigotes, while LdPAT4 was undetected in the amastigote form (Supplementary Table 1). These findings indicate profound palmitoylation in the promastigote stage that has been further explored.

### Gene ontology and functional analysis revealed enriched clusters of motility associated proteins

Recent reports on palmitoylation in Trypanosomatids like *T. brucei* have outlined their probable roles in intracellular trafficking and parasite motility. In this direction, we further elucidated the mechanism and implications of palmitoylation in the promastigotes. The expressed proteins of this stage were analyzed for significant GO categories and further delineated into different modules based on their cellular and molecular functions. GO terms including catalytic activity, metabolic processes, cell motility, molecular function regulator, and microtubule were found to be enriched (Figure 3B).

**Figure 4:**
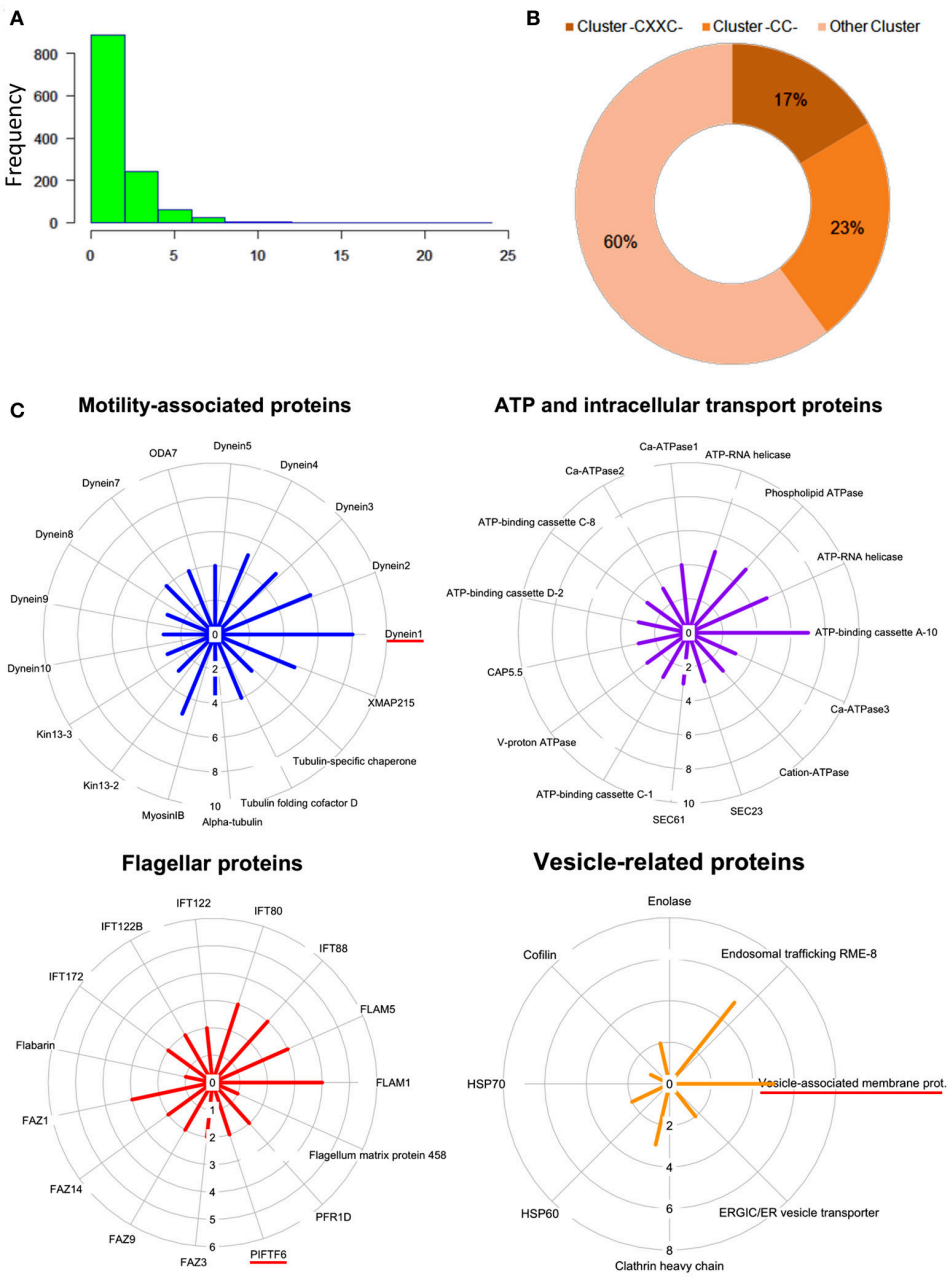
**(A)** Histogram plot showing frequency of palmitoylation in all predicted palmitoylated proteins. **(B)** Representative doughnut plots showing the prevalence of three categories of predicted palmitoylated site clusters. **(C)** Circular plots showing shortlisted novel palmitoylated protein modules and their respective protein palmitoylation rates. The bar length in the circular plot indicates the protein expression level.

To evaluate the frequency of palmitoylation in promastigote-specific proteins, we have also identified the different Cysteine-rich palmitoylation sites using CSS-Palm and cataloged the frequency profiles of highly palmitoylated proteins. The results delineated a maximum number of 23 sites in an unannotated protein, LdBPK_355080.1 out of 1225 proteins (Figures 4A,B). Precisely, the highest numbers of proteins were found to have 2–4 palmitoylated sites (Figure 4A). On categorizing the palmitoylation motifs based on CSS-Palm (I-III), we found that 23% of proteins fall under Cluster I (-CC-) category, 17% under Cluster II (-CXXC-) and the rest (60%) fall under the type III category (Figure 4B). The above data strongly indicated the role of palmitoylation in motility, endocytosis and other essential biological processes.

Following GO analysis and further annotation of all 1225 genes, 4 primary functional modules were unraveled namely; ATP and intracellular transport, vesicle, cellular motility and flagella associated proteins (Figure 4B). Prediction analysis of these functional modules revealed crucial proteins associated with the para-flagellar rod function like FLAM1, FLAM5 and other flagellar motor assembly proteins including Dynein1-5 (Figure 4C). Previously in other eukaryotic parasites like *T. gondii* and *P. falciparum*, palmitoylation has been observed to regulate motility of the parasite, thus affecting its overall survival (Alonso et al., 2012; Frénal et al., 2013; Wetzel et al., 2015). Therefore, it is assumed that proteins present in the motility and flagella associated modules might be essential for promastigote cell survival.

### Attenuation of global palmitoylation led to altered acylation followed by distorted morphology in promastigotes

**Figure 5:**
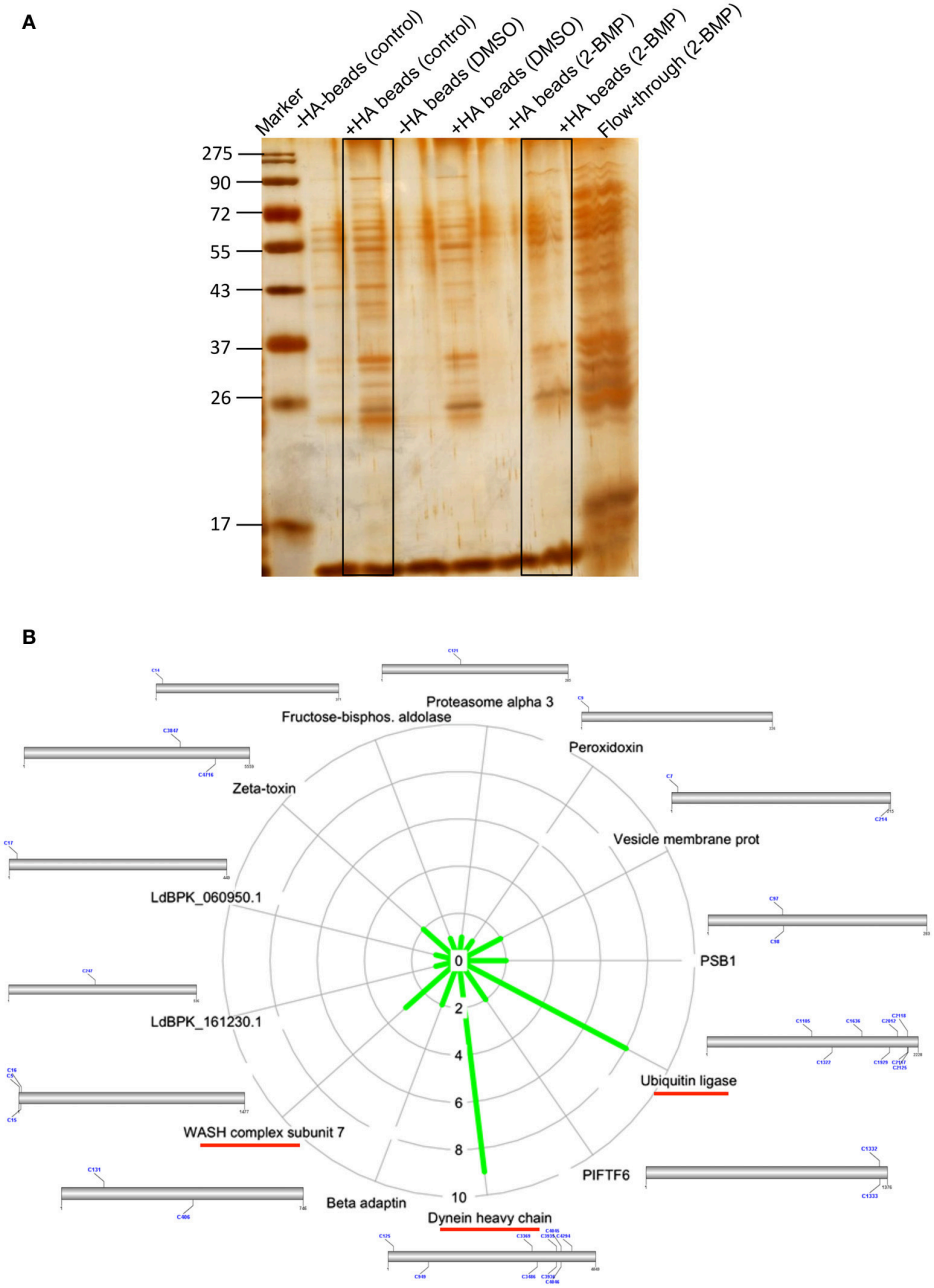
**(A)** Silver staining of protein elutes purified via Acyl Biotin Exchange method. **(B)** Identification of overlapping list of palmitoylated proteins with *in-silico* palmitoylome, along with the display of palmitoylated Cysteine sites.

Inhibition of the active palmitoylation process in *L. donovani* using 2-BMP resulted in profound cytotoxicity as well as inhibitory effects at the nanomolar range in LDH and MTT assays respectively (Supplementary Figures 5A,B). The IC_50_ value of 2-BMP was recorded to be 750 nM (Supplementary Figure 5B). To further characterize the impact of 2-BMP on the global palmitoylome of *L. donovani*, we have performed Acyl-biotin exchange of promastigote-specific proteins from the whole cell lysate. The results have confirmed the substantial reduction in palmitoylated protein pools when treated with 2-BMP in hydroxylamine treated lysates (Figure 5A). The individual lysates were assessed quantitatively using LC-MS. Upon analysis of strongly palmitoylated (+HA-control) samples and analysis of individual gel based elutes of lanes (+HA-lane no. 2 and 6), we observed an overlapping list of proteins with the prior *in-silico* predicted pool of palmitoylated proteins (Figures 4C, 5). This list encompassed several essential palmitoylated proteins like WASH complex subunit protein, dynein heavy chain, ubiquitin ligase, etc. which were further analyzed for the number of palmitoylated sites per protein (Figure 5B, Supplementary Figure 6A). The pre-cleared and crude extracts preceding ABE purification have been demonstrated in Supplementary Data (Supplementary Figure 6B).

**Figure 6:**
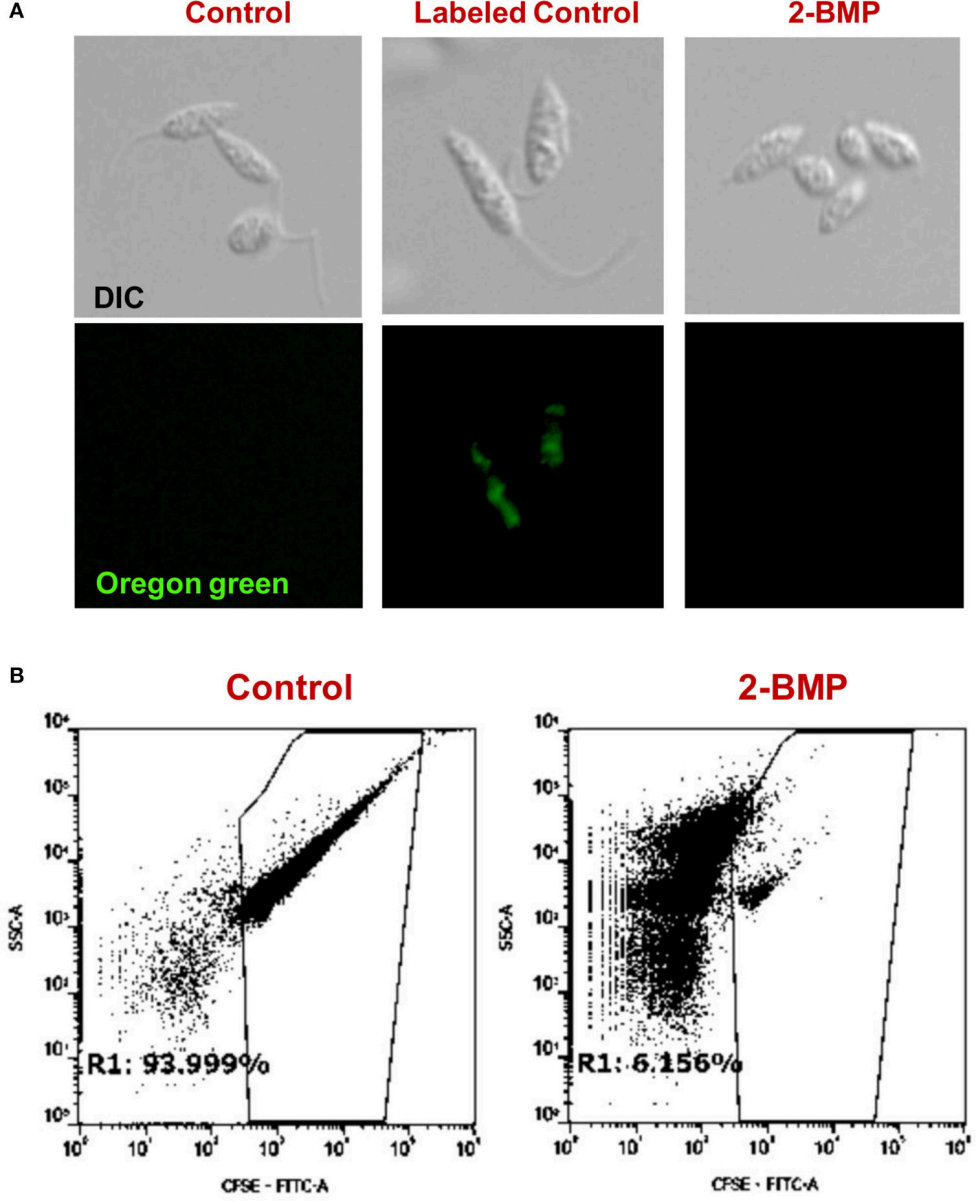
**(A)** Clickable metabolic labeling of the promastigotes following 2-BMP treatment show weak palmitoylation profiles in comparison to untreated parasites. **(B)** Representative dot plots showing a pronounced reduction in palmitoylation following 2-BMP treatment.

To identify alterations in the total palmitoylome, we employed a global protein palmitoylation reporter assay using a robust, two-step labeling clickable chemistry method (Wang et al., 2003). The immunofluorescence assay showed a pronounced reduction in green fluorescence of Oregon Green-488 in 2-BMP treated samples as compared to Control, which strongly suggests altered protein palmitoylation following 2-BMP treatment (Figure 6A). This also emphasizes the role of palmitoylation in the growth and proliferation of *L. donovani*. The parasite morphology was also seen to be perturbed upon 2-BMP treatment with the cells rounding up and evident flagellar loss. Flow cytometry analysis substantiated the visible microscopy data, which is evident by 94% stained cells in control, and exponential reduction in staining on 2-BMP treatment (6%) (Figure 6B). Overall, these findings suggest that protein palmitoylation is crucial for promastigote cell morphology and survival.

### Perturbed palmitoylation is linked to reduced parasite invasion efficiency and motility

**Figure 7:**
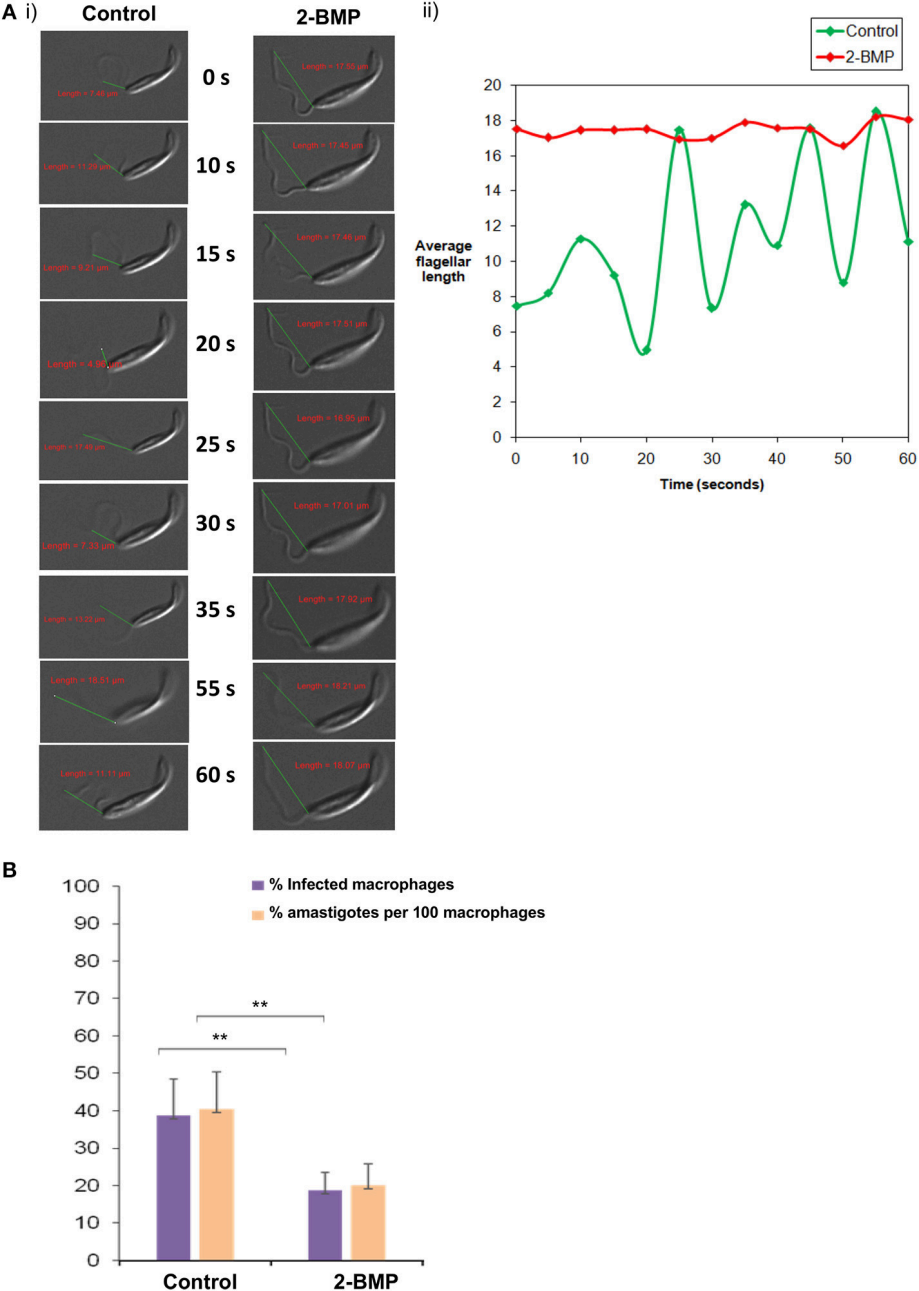
**(A)** Effects on flagellar beating upon inhibition of palmitoylation. **(i)** Time-lapse video microscopy showed detrimental effects on flagellar motility upon treatment with 2-BMP inhibitor. The scale indicates 5 μm. **(ii)** The graph shows linear, attenuated waveform for 2-BMP treated flagella, while untreated flagella displayed healthy beating with unattenuated waveforms. **(B)** Quantitative estimation of amastigote counts per 100 macrophages following 2-BMP treatment was used for evaluating host invasion efficiency (**p < < 0.05). Data are represented as mean ± SEM.

Specifically, the effect on flagellar motility was assessed by quantification of the flagellar beat parameters. In case of control promastigotes, the flagellar waveform was found to be symmetrical and originated from the tip of the flagella (Figures 7Ai,ii). The 2-BMP treated parasites showed a loss of symmetry in the waveform with restriction in movement (Figures 7Ai,ii). In individual flagellum, the wavelength (tip to base length) was found to be highly variable during 0–60 s of video recording in normally conditioned parasites, whereas in the treated parasites it was observed to be mostly static (Figure 7Ai). Calculation of average flagella beat speed revealed substantially reduced beating in case of 2-BMP treated parasites (0.094 μm/s) when compared to control parasites (1.22 μm/s) (Supplementary Table 4).

We also calculated the parasite load in macrophages following infection with promastigotes (Parasite load = % amastigote/100 macrophages). We observed ~50% reduction in parasitemia upon palmitoylation repression whereas, the uninfected parasites remained invasive (40%) (Figure 7B). These critical findings conclude that palmitoylation plays crucial roles in motility, especially flagellar movement and overall invasion rate of promastigotes into host macrophage cells.

## Discussion

Leishmania belongs to the family of trypanosomatids which represents a unique model system to study protein acylation mechanisms underlying PTMs like palmitoylation (Goldston et al., 2014). This is a biochemical reaction which involves the enzymatic modification of Cysteine residues by PATs thus, resulting in proper protein folding and cellular homeostasis. Since differential expression of PATs is strongly parasite stage-specific, it is imperative to investigate their localization, expression and functional implications for better understanding of disease progression. Earlier findings from Trypanosomatids have shown that protein modifications including palmitoylation and myristoylation play critical roles in intracellular trafficking important for flagellar activity (Brannigan et al., 2010; Goldston et al., 2014; Wright et al., 2015). It is noteworthy that sequence/structural annotation and functional characterization in case of *L. donovani* are still in their infancy. In this study, we have combined *in-silico* proteomics, biochemical assays and *in-vitro* parasite based studies to ascertain novel Leishmania palmitoyl acyltransferases and explore their roles in the parasite life cycle for future development of chemotherapeutics.

Few burning questions reflect on the potential roles of PATs in post-translational regulation of *L. donovani* and their applications as putative drug targets for Visceral Leishmaniasis. To emphasize, the inter-species structural differences in PAT enzymes, specifically between *Leishmania* and human species should be exploited for designing effective anti-Leishmanial drugs. To explore the above possibilities, we for the first time have annotated 20 DHHC domain-containing protein sequences in *L. donovani* (LdPATs) and elucidated the potential role of global palmitoylation in parasite growth and survival. We further individually characterized the promastigote-specific palmitoyl acyltransferase 4 (LdPAT4) as well as evaluated its activity and cellular localization in the parasite. This is the first report to establish the significance of palmitoylation in the biology of *Leishmania* parasite and its interaction with macrophages. Through *in-silico* analyses, we have ascertained the palmitoylated proteome of *L. donovani*, out of which the enriched protein clusters displayed crucial roles in cell survival, motility, signaling, etc. The deconvoluted protein modules represented the differential expression of specific proteins in motile as well as non-motile forms of the parasite. The significant protein modules were comprised of two crucial motility-associated protein clusters (Dynein family and FLAM family) as well as vesicle and intracellular transport proteins. To highlight, dynein protein family is associated with the flagella assembly and motor functions, whereas the FLAM family constitutes the structural proteins inside the flagellar apparatus which includes the flagellar pocket and para-flagellar rod.

**Figure 8:**
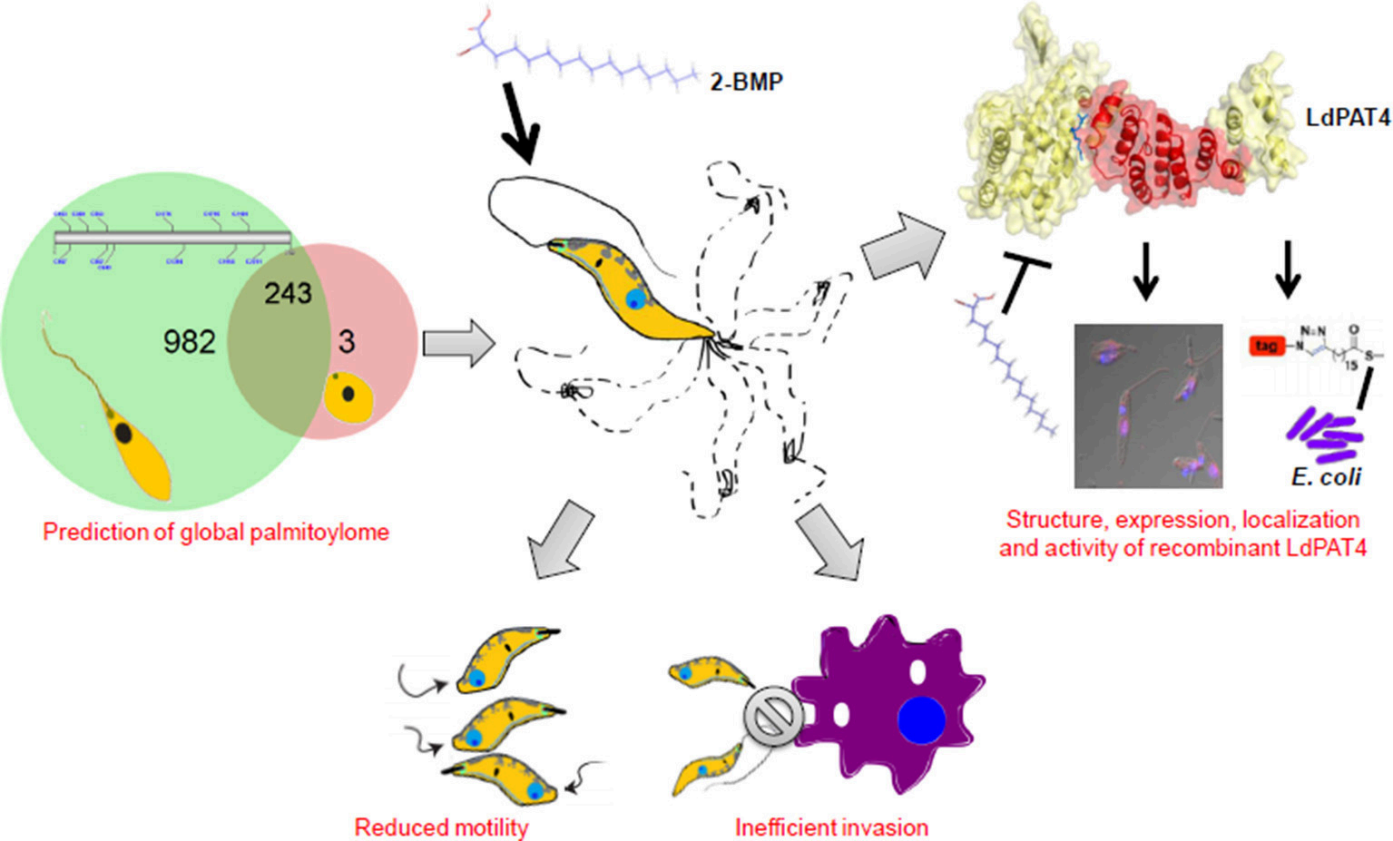
Schematic model illustrating the role of palmitoylation in *L. donovani* biology.

To elucidate the impact of palmitoylation on parasite motility and invasion, we have performed *in-vitro* assays using one of the specific inhibitors of dynamic palmitoylation (2-BMP). Following treatment with 2-BMP, palmitoylation was found to be drastically affected in metabolically labeled promastigotes as observed in the clickable probe-based experiment. The findings have established a direct link of palmitoylation with parasite flagella motility and invasion as evidenced by significant loss of movement and perturbed invasion (Figure 7, Supplementary Videos 1, 2). Our data has cataloged 20 LdPATs, among which LdPAT4 is found to be highly expressed in promastigotes and undetected in the amastigotes. Cloning and expression of LdPAT4-DHHC have validated a ~40 kDa protein as per the annotated size. To authenticate the acyltransferase activity of LdPAT4-DHHC, we have used a novel strategy which involves a clickable chemistry-based experiment in the bacterial system (*E. coli*) which is representative of a PTM-null model. The findings authenticated catalytic activity of LdPAT4-DHHC as shown by predominant incorporation of palmitic acid analogs into transformed *E. coli* (Figure 2C). Furthermore, functional characterization of LdPAT4-DHHC showed its pronounced localization in the parasite flagellar pocket indicating its plausible function in *L. donovani* motility as well as endocytosis. In summary, our overall hypothesis delineated *Leishmania*-specific PATs, established the significance of palmitoylation in *L. donovani*, and proposes LdPAT4 as a novel drug target that can pave the way for designing efficacious chemotherapeutics for Leishmaniasis (Figure 8).

## Significance

Leishmaniasis afflicts millions of people in the tropics and still awaits successful pharmacological interventions. It is imperative to employ new biochemical approaches for identification of potential biomarkers and development of efficacious chemotherapeutics. Palmitoylation, a lipid-based post-translational modification is known to regulate biological processes of eukaryotic parasites. However, this process was poorly understood in *L. donovani*. Herein, we have employed an *in-silico* biology-coupled chemical proteomics approach to gain a macroscopic view of global palmitoylation and underpin its role in parasite motility and life cycle progression. Our study established a catalog of DHHC signature domain containing PATs, predicted the global palmitoylome of *L. donovani* revealing druggable targets and therefore, reinstated the role of palmitoylation in parasite biology. This work also reports for the first time a unique engineered prokaryotic system for *ex-vivo* characterization of PAT activity that can be further explored for high-throughput drug screening. Overall, this work constitutes a global combinatorial (*in-silico* and *in-vitro*) analysis of palmitoylation in *L. donovani* and thus, contributes to the fundamental understanding of palmitoylation and its druggability.

## Author contributions

SS conceptualized, designed all the experiments, and analyzed the data. RA and SP planned the computational strategy. RA performed all computational analyses and mass spec data analysis. DR, RA, and SG performed click chemistry and motility assays. PY and RK characterized the recombinant protein and performed click chemistry in *E. coli*. RK and PY executed the Acyl-biotin exchange purification and performed microscopy. RA, PY, DR, and SS analyzed the data, and RA, SP, and SS wrote the manuscript with analytical input from SS.

### Conflict of interest statement

The authors declare that the research was conducted in the absence of any commercial or financial relationships that could be construed as a potential conflict of interest.

## Supplementary material

Supplementary Figure 1 Schematic overview of *in-silico* strategy employed to predict the global palmitoylome of *L. donovani*.

Supplementary Figure 2 Semi-quantitative PCR analysis showing annotated LdPATs along with several validated housekeeping genes like ATPase, promastigote-specific gene LACK and probable molecular targets of palmitoylation including the FLAM and Dynein family of genes.

Supplementary Figure 3 **(A)** Inter-species phylogenetic analysis showing strong conservation across varied eukaryotic parasite gene families of *T. brucei, L. donovani, P. falciparum* and *H. sapiens*. **(B)** Intra-species phylogenetic bubble-sorted tree showing strong clustering amongst species within the *Leishmania* genus, as shown in LdPAT4 and LdPAT6 clusters. Size of the bubble indicates the confidence score of each branch node in the tree.

Supplementary Figure 4 **(A)** Validation of recombinant LdPAT4-DHHC using SDS-PAGE based analysis. Coomassie brilliant blue staining showing expression of the recombinant LdPAT4-DHHC protein in elute fraction 3. **(B)** SDS-PAGE analysis followed by western blotting demonstrates expression of ~40 kDa LdPAT4 protein.

Supplementary Figure 5 **(A)** Cytotoxicity analysis using LDH calorimetric assay following 2-BMP treatment of promastigotes. **(B)** Determination of IC_50_ concentration for 2-BMP inhibitor using MTT assay.

Supplementary Figure 6 **(A)** Top palmitoylated proteins displaying no. of palmitoylated Cysteine sites ranging from 6 to 23. **(B)** Silver-stained gel showing the pre-cleared bands and crude extract from ABE technique.

Supplementary Table 1 LdPATs expressed in prominent stages of L. donovani. ^*^Samples are A-Amastigote, P-Promastigote.

Supplementary Table 2 RT-PCR analysis showing relative expression w.r.t housekeeping controls, actin (ΔCT). NOTE: Separate actin controls were maintained for all 3 gels.

Supplementary Table 3 Primer sequences of 20 LdPATs, promastigote controls, actin gene and some potentially palmitoylated proteins. FP-Forward primer, RP-Reverse primer

Supplementary Table 4 Average beating speed of flagella (μm/s) calculated of untreated promastigotes and promastigotes treated with 2-BMP.

Supplementary Video 1 Time lapse video microscopy showing active, motile parasites which were left untreated.

Supplementary Video 2 Time lapse video microscopy showing detrimental effects on parasite motility following 2-BMP treatment.

## References

[B1] Alonso A. M. Coceres V. M. De Napoli M. G. Nieto Guil A. F. Angel S. O. Corvi M. M. (2012). Protein palmitoylation inhibition by 2-bromopalmitate alters gliding, host cell invasion and parasite morphology in Toxoplasma gondii. Mol. Biochem. Parasitol. 184, 39–43. 10.1016/j.molbiopara.2012.03.006 22484029 PMC3358525

[B2] Aslett M. Aurrecoechea C. Berriman M. Brestelli J. Brunk B. P. Carrington M.. (2010). TriTrypDB: a functional genomic resource for the Trypanosomatidae. Nucleic Acids Res. 38, D457–D462. 10.1093/nar/gkp851 19843604 PMC2808979

[B3] Aurrecoechea C. Brestelli J. Brunk B. P. Dommer J. Fischer S. Gajria B.. (2009). PlasmoDB: a functional genomic database for malaria parasites. Nucleic Acids Res. 37, D539–D543. 10.1093/nar/gkn814 18957442 PMC2686598

[B4] Brannigan J. A. Smith B. A. Yu Z. Brzozowski A. M. Hodgkinson M. R. Maroof A.. (2010). N-myristoyltransferase from *Leishmania donovani*: structural and functional characterisation of a potential drug target for visceral leishmaniasis. J. Mol. Biol. 396, 985–999. 10.1016/j.jmb.2009.12.032 20036251 PMC2829124

[B5] Caballero M. C. Alonso A. M. Deng B. Attias M. De Souza W. Corvi M. M. (2016). Identification of new palmitoylated proteins in Toxoplasma gondii. Biochim. Biophys. Acta 1864, 400–408. 10.1016/j.bbapap.2016.01.010 26825284 PMC4857766

[B6] Delano W. L. (2002). The PyMOL Molecular Graphics System. Available online at: http://pymol.org/academic.

[B7] Draper J. M. Smith C. D. (2010). DHHC20: a human palmitoyl acyltransferase that causes cellular transformation. Mol. Membr. Biol. 27, 123–136. 10.3109/09687681003616854 20334580 PMC2893407

[B8] Florens L. Washburn M. P. Raine J. D. Anthony R. M. Grainger M. Haynes J. D.. (2002). A proteomic view of the *Plasmodium falciparum* life cycle. Nature 419, 520–526. 10.1038/nature01107 12368866

[B9] Foe I. T. Child M. A. Majmudar J. D. Krishnamurthy S. Van Der Linden W. A. Ward G. E.. (2015). Global Analysis of palmitoylated proteins in *Toxoplasma gondii*. Cell Host Microbe 18, 501–511. 10.1016/j.chom.2015.09.006 26468752 PMC4694575

[B10] Fra A. Yoboue E. D. Sitia R. (2017). Cysteines as redox molecular switches and targets of disease. Front. Mol. Neurosci. 10:167. 10.3389/fnmol.2017.00167 28634440 PMC5459893

[B11] Frénal K. Tay C. L. Mueller C. Bushell E. S. Jia Y. Graindorge A.. (2013). Global analysis of apicomplexan protein S-Acyl transferases reveals an enzyme essential for invasion. Traffic 14, 895–911. 10.1111/tra.12081 23638681 PMC3813974

[B12] Goldston A. M. Sharma A. I. Paul K. S. Engman D. M. (2014). Acylation in trypanosomatids: an essential process and potential drug target. Trends Parasitol. 30, 350–360. 10.1016/j.pt.2014.05.003 24954795 PMC4190163

[B13] Huerta-Cepas J. Serra F. Bork P. (2016). ETE 3: reconstruction, analysis, and visualization of phylogenomic data. Mol. Biol. Evol. 33, 1635–1638. 10.1093/molbev/msw046 26921390 PMC4868116

[B14] Jones M. L. Collins M. O. Goulding D. Choudhary J. S. Rayner J. C. (2012). Analysis of protein palmitoylation reveals a pervasive role in Plasmodium development and pathogenesis. Cell Host Microbe 12, 246–258. 10.1016/j.chom.2012.06.005 22901544 PMC3501726

[B15] Kall L. Krogh A. Sonnhammer E. L. (2007). Advantages of combined transmembrane topology and signal peptide prediction–the Phobius web server. Nucleic Acids Res. 35, W429–W432. 10.1093/nar/gkm256 17483518 PMC1933244

[B16] Leroux A. E. Krauth-Siegel R. L. (2016). Thiol redox biology of trypanosomatids and potential targets for chemotherapy. Mol. Biochem. Parasitol. 206, 67–74. 10.1016/j.molbiopara.2015.11.003 26592324

[B17] Letunic I. Copley R. R. Schmidt S. Ciccarelli F. D. Doerks T. Schultz J.. (2004). SMART 4.0: towards genomic data integration. Nucleic Acids Res. 32, D142–D144. 10.1093/nar/gkh088 14681379 PMC308822

[B18] Mitchell A. Chang H.-Y. Daugherty L. Fraser M. Hunter S. Lopez R.. (2015). The InterPro protein families database: the classification resource after 15 years. Nucleic Acids Res. 43, D213–D221. 10.1093/nar/gku1243 25428371 PMC4383996

[B19] Mitchell D. A. Vasudevan A. Linder M. E. Deschenes R. J. (2006). Protein palmitoylation by a family of DHHC protein S-acyltransferases. J. Lipid Res. 47, 1118–1127. 10.1194/jlr.R600007-JLR200 16582420

[B20] Morris G. M. Huey R. Lindstrom W. Sanner M. F. Belew R. K. Goodsell D. S.. (2009). AutoDock4 and AutoDockTools4: automated docking with selective receptor flexibility. J. Comput. Chem. 30, 2785–2791. 10.1002/jcc.21256 19399780 PMC2760638

[B21] Nirujogi R. S. Pawar H. Renuse S. Kumar P. Chavan S. Sathe G.. (2014). Moving from unsequenced to sequenced genome: reanalysis of the proteome of *Leishmania donovani*. J. Proteomics 97, 48–61. 10.1016/j.jprot.2013.04.021 23665000 PMC4710096

[B22] Ren J. Wen L. Gao X. Jin C. Xue Y. Yao X. (2008). CSS-Palm 2.0: an updated software for palmitoylation sites prediction. Protein Eng. Design Select. 21, 639–644. 10.1093/protein/gzn039 18753194 PMC2569006

[B23] Santos J. M. Kehrer J. Franke-Fayard B. Frischknecht F. Janse C. J. Mair G. R. (2015). The plasmodium palmitoyl-S-acyl-transferase DHHC2 is essential for ookinete morphogenesis and malaria transmission. Sci. Rep. 5:16034. 10.1038/srep16034 26526684 PMC4630622

[B24] Tamura K. Stecher G. Peterson D. Filipski A. Kumar S. (2013). MEGA6: molecular evolutionary genetics analysis version 6.0. Mol. Biol. Evol. 30, 2725–2729. 10.1093/molbev/mst197 24132122 PMC3840312

[B25] Team R. (2015). RStudio: Integrated Development for R. Boston, MA: RStudio, Inc.

[B26] Trott O. Olson A. J. (2010). AutoDock Vina: improving the speed and accuracy of docking with a new scoring function, efficient optimization, and multithreading. J. Comput. Chem. 31, 455–461. 10.1002/jcc.21334 19499576 PMC3041641

[B27] Untergasser A. Nijveen H. Rao X. Bisseling T. Geurts R. Leunissen J. A. (2007). Primer3Plus, an enhanced web interface to Primer3. Nucleic Acids Res. 35, W71–W74. 10.1093/nar/gkm306 17485472 PMC1933133

[B28] Wan J. Roth A. F. Bailey A. O. Davis N. G. (2007). Palmitoylated proteins: purification and identification. Nat. Protoc. 2, 1573–1584. 10.1038/nprot.2007.225 17585299

[B29] Wang Q. Chan T. R. Hilgraf R. Fokin V. V. Sharpless K. B. Finn M. G. (2003). Bioconjugation by copper (I)-catalyzed azide-alkyne [3 + 2] cycloaddition. J. Am. Chem. Soc. 125, 3192–3193. 10.1021/ja021381e 12630856

[B30] Wastling J. M. Armstrong S. D. Krishna R. Xia D. (2012). Parasites, proteomes and systems: has Descartes' clock run out of time? Parasitology 139, 1103–1118. 10.1017/S0031182012000716 22828391 PMC3428110

[B31] Waterhouse A. M. Procter J. B. Martin D. M. A. Clamp M. Barton G. J. (2009). Jalview Version 2—a multiple sequence alignment editor and analysis workbench. Bioinformatics 25, 1189–1191. 10.1093/bioinformatics/btp033 19151095 PMC2672624

[B32] Wetzel J. Herrmann S. Swapna L. S. Prusty D. John Peter A. T. Kono M.. (2015). The role of palmitoylation for protein recruitment to the inner membrane complex of the malaria parasite. J. Biol. Chem. 290, 1712–1728. 10.1074/jbc.M114.598094 25425642 PMC4340414

[B33] Wright M. H. Paape D. Storck E. M. Serwa R. A. Smith D. F. Tate E. W. (2015). Global analysis of protein N-myristoylation and exploration of N-myristoyltransferase as a drug target in the neglected human pathogen *Leishmania donovani*. Chem. Biol. 22, 342–354. 10.1016/j.chembiol.2015.01.003 25728269 PMC4372256

[B34] Xu D. Zhang Y. (2011). Improving the physical realism and structural accuracy of protein models by a two-step atomic-level energy minimization. Biophys. J. 101, 2525–2534. 10.1016/j.bpj.2011.10.024 22098752 PMC3218324

[B35] Zhang Y. (2008). I-TASSER server for protein 3D structure prediction. BMC Bioinformatics 9:40. 10.1186/1471-2105-9-40 18215316 PMC2245901

