# Supplementary figures and images for: Identification and Characterization of a Novel Palmitoyl Acyltransferase as a Druggable Rheostat of Dynamic Palmitoylome in L. donovani
Source: Front Cell Infect Microbiol. 2018 Jun 20;8:186. doi: 10.3389/fcimb.2018.00186 (PMC6022219; doi:10.3389/fcimb.2018.00186)

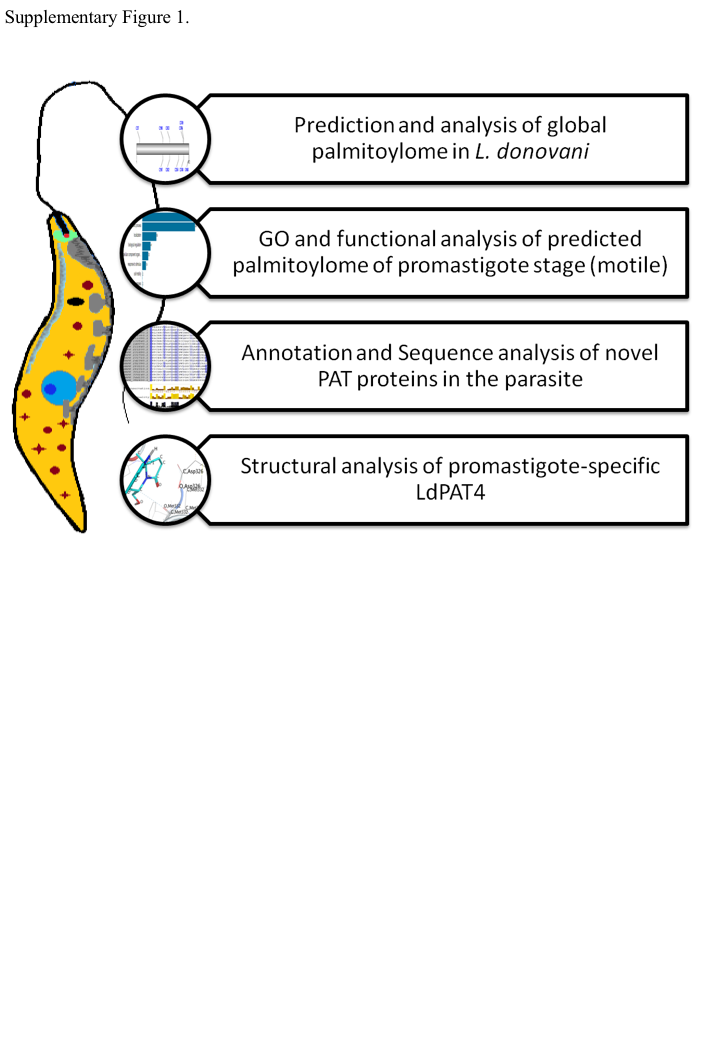

Supplement: Supplementary Figure 1 — Schematic overview of in-silico strategy employed to predict the global palmitoylome of L. donovani. [file Image_1.TIFF]

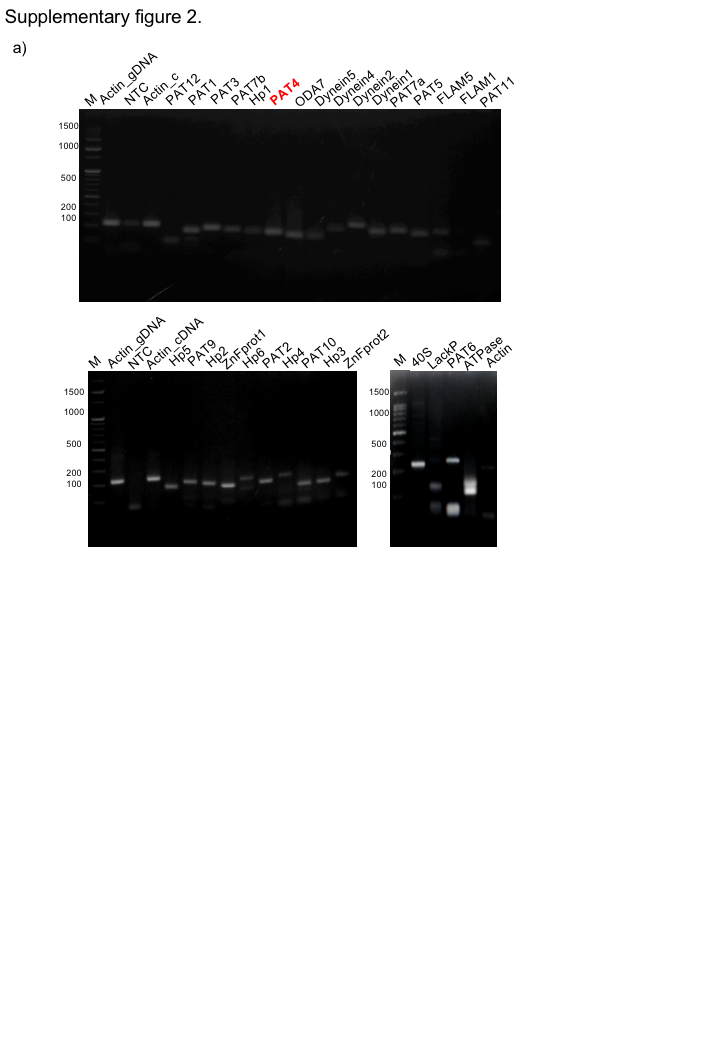

Supplement: Supplementary Figure 2 — Semi-quantitative PCR analysis showing annotated LdPATs along with several validated housekeeping genes like ATPase, promastigote-specific gene LACK and probable molecular targets of palmitoylation including the FLAM and Dynein family of genes. [file Image_2.TIFF]

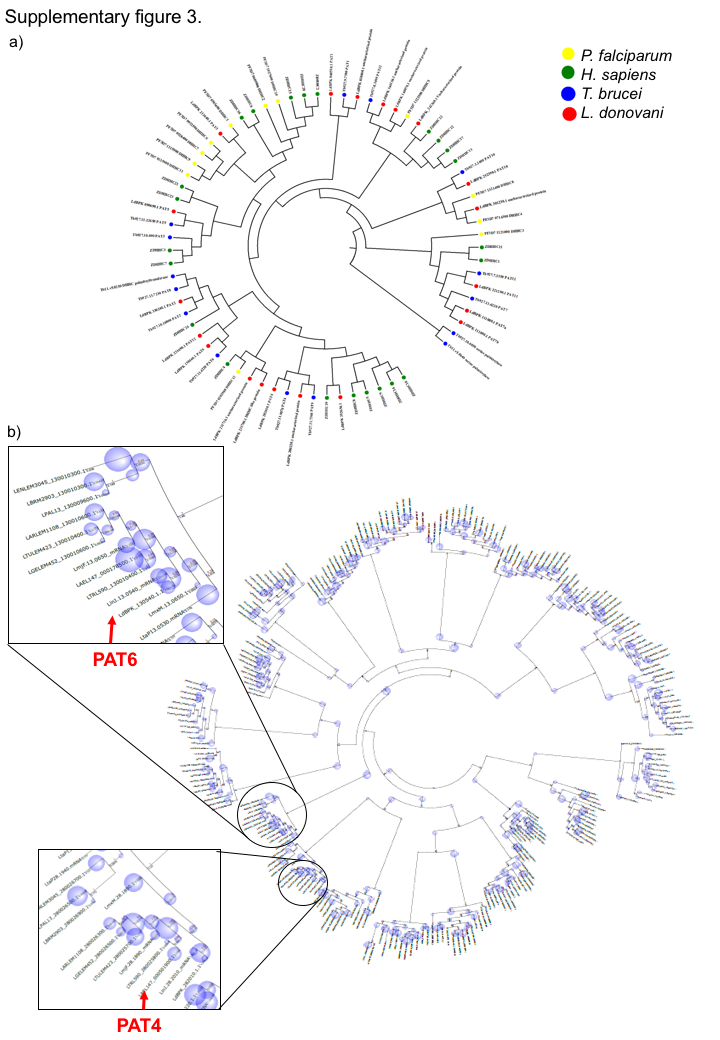

Supplement: Supplementary Figure 3 — (A) Inter-species phylogenetic analysis showing strong conservation across varied eukaryotic parasite gene families of T. brucei, L. donovani, P. falciparum and H. sapiens. (B) Intra-species phylogenetic bubble-sorted tree showing strong clustering amongst species within the Leishmania genus, as shown in LdPAT4 and LdPAT6 clusters. Size of the bubble indicates the confidence score of each branch node in the tree. [file Image_3.TIFF]

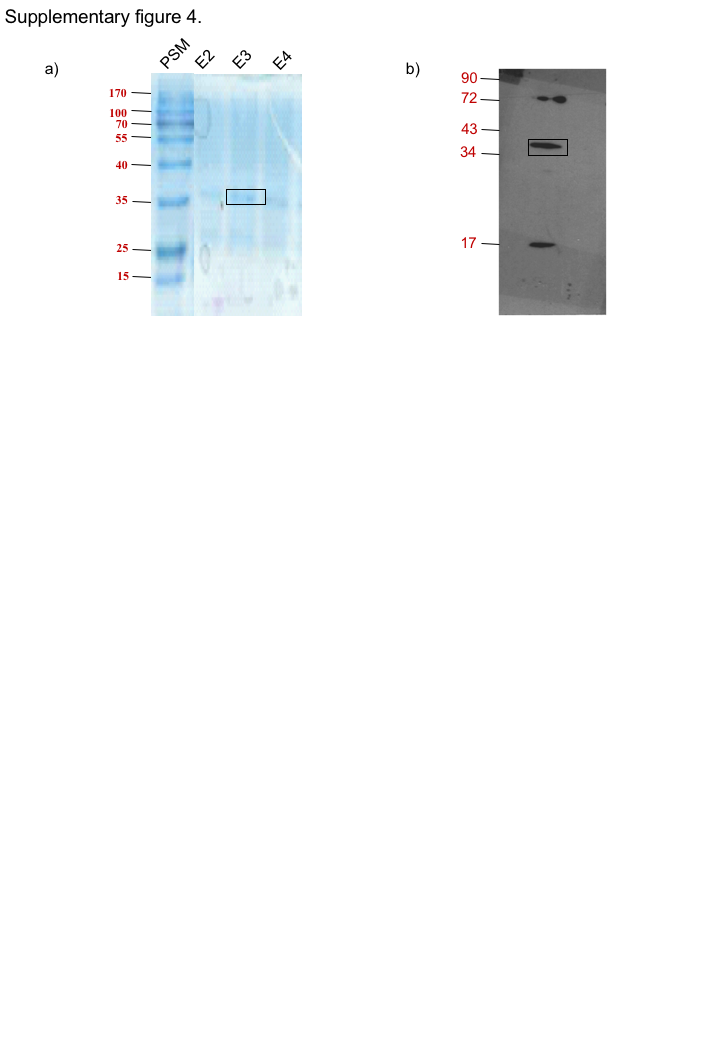

Supplement: Supplementary Figure 4 — (A) Validation of recombinant LdPAT4-DHHC using SDS-PAGE based analysis. Coomassie brilliant blue staining showing expression of the recombinant LdPAT4-DHHC protein in elute fraction 3. (B) SDS-PAGE analysis followed by western blotting demonstrates expression of ~40 kDa LdPAT4 protein. [file Image_4.TIFF]

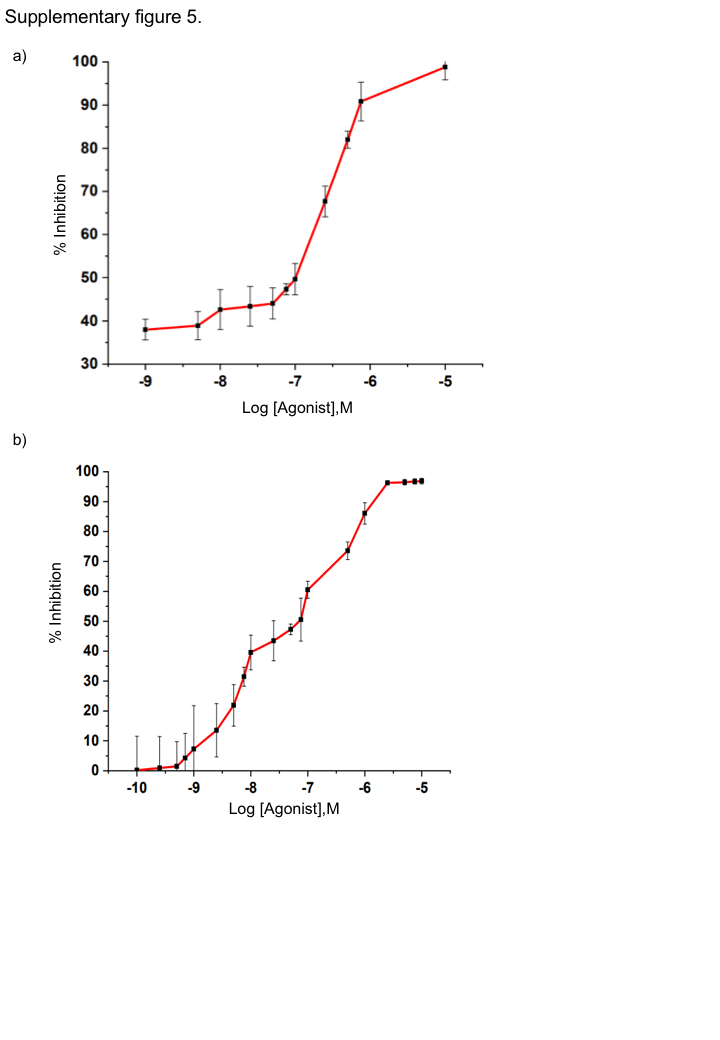

Supplement: Supplementary Figure 5 — (A) Cytotoxicity analysis using LDH calorimetric assay following 2-BMP treatment of promastigotes. (B) Determination of IC50 concentration for 2-BMP inhibitor using MTT assay. [file Image_5.TIFF]

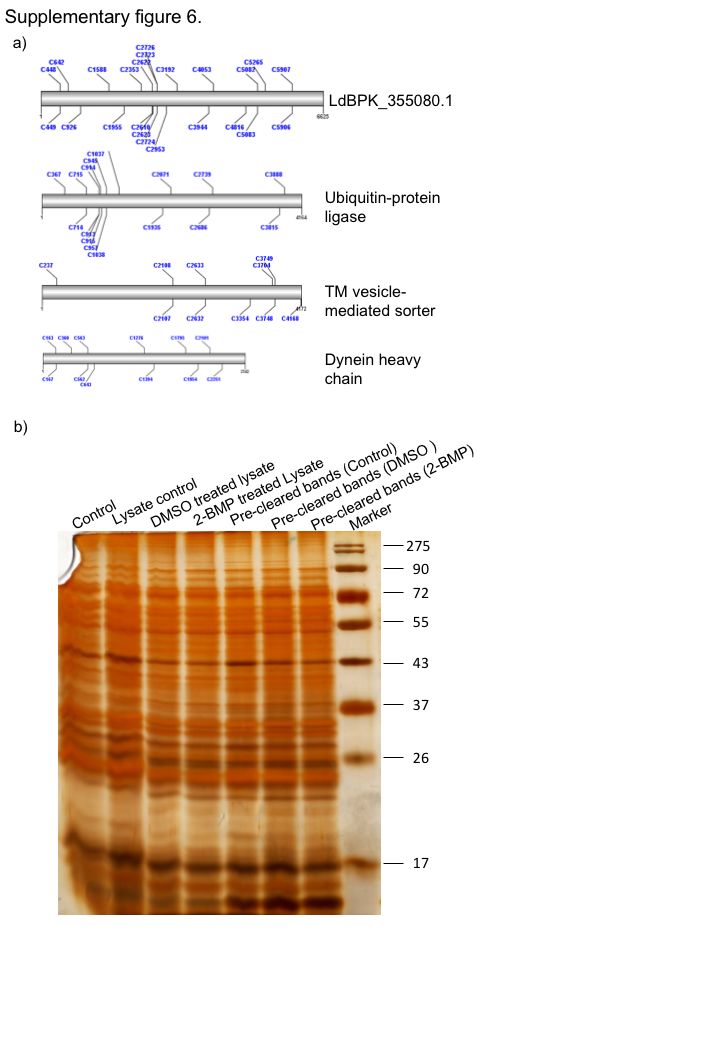

Supplement: Supplementary Figure 6 — (A) Top palmitoylated proteins displaying no. of palmitoylated Cysteine sites ranging from 6 to 23. (B) Silver-stained gel showing the pre-cleared bands and crude extract from ABE technique. [file Image_6.TIFF]
