# Supplementary material for: Identification and Characterization of a Novel Palmitoyl Acyltransferase as a Druggable Rheostat of Dynamic Palmitoylome in L. donovani
Source: Front Cell Infect Microbiol. 2018 Jun 20;8:186. doi: 10.3389/fcimb.2018.00186 (PMC6022219; doi:10.3389/fcimb.2018.00186)
Supplement: Supplementary Table 1 — LdPATs expressed in prominent stages of L. donovani. *Samples are A-Amastigote, P-Promastigote. [file Data_Sheet_1.DOC]

Supplementary data

| **Gene ID** | **Sample*** | | **Sequences** | | **Spectra** | |
| --- | --- | --- | --- | --- | --- | --- |
|  | A | P | A | P | A | P |
| LdBPK_282010.1/ PAT4 | NO | YES | 0 | 2 | 0 | 9 |
| LdBPK_130540.1/ PAT6 | YES | YES | 2 | 5 | 35 | 44 |

**Suppl. Table 1.** LdPATs expressed in prominent stages of *L. donovani*. * Samples are A- Amastigote, P- Promastigote**.**

| GEL 1 | | | |  | GEL 2 | | | |
| --- | --- | --- | --- | --- | --- | --- | --- | --- |
| **SAMPLE** | **TARGET** | **ΔCт** | **SD** |  | **SAMPLE** | **TARGET** | **ΔCт** | **SD** |
| gDNA | ACTIN | -4.34971 | 0.03 |  | gDNA | ACTIN | -7.65649 | 0.04 |
| NTC | ACTIN | 10.45141 | 0.03 |  | NTC | ACTIN | 18.59099 | 0.10 |
| cDNA | ACTIN | 0 | 0.26 |  | cDNA | ACTIN | 0 | 0.10 |
| cDNA | PAT12 | 4.56128 | 0.26 |  | cDNA | Hp5 | 6.562179 | 0.21 |
| cDNA | PAT1 | 7.17004 | 0.08 |  | cDNA | PAT9 | 7.139713 | 0.21 |
| cDNA | PAT3 | 6.28115 | 0.08 |  | cDNA | Hp2 | 10.74437 | 0.56 |
| cDNA | PAT7b | 8.59332 | 0.12 |  | cDNA | ZnFprot1 | 9.163127 | 0.56 |
| cDNA | Hp1 | 6.55259 | 0.12 |  | cDNA | Hp6 | 9.987714 | 0.02 |
| cDNA | PAT4 | 6.37736 | 1.15 |  | cDNA | PAT2 | 7.4 | 0.02 |
| cDNA | ODA7 | 4.36794 | 1.15 |  | cDNA | Hp4 | 12.4 | 0.42 |
| cDNA | Dynein5 | 1.97187 | 4.83 |  | cDNA | PAT10 | 1.4 | 0.42 |
| cDNA | Dynein4 | 5.59645 | 4.83 |  | cDNA | Hp3 | 7 | 0.63 |
| cDNA | Dynein2 | 2.70108 | 0.05 |  | cDNA | ZnFprot2 | 5.6 | 0.63 |
| cDNA | Dynein1 | 6.54344 | 0.05 |  |  |  |  |  |
| cDNA | PAT7a | 4.04778 | 0.81 |  | GEL 3 | | | |
| cDNA | PAT5 | 5.56219 | 0.81 |  | **SAMPLE** | **TARGET** | **ΔCт** | **SD** |
| cDNA | FLAM5 | 2.5062 | 0.54 |  | gDNA | ACTIN | -8.65649 | 2.14 |
| cDNA | FLAM1 | 1.46145 | 0.54 |  | NTC | ACTIN | 17.59099 | 2.14 |
| cDNA | PAT11 | 5.53555 | 0.04 |  | cDNA | ACTIN | 0 | 0.05 |
|  |  |  |  |  | cDNA | 40S ribosomal subunit | 4.47222 | 0.05 |
|  |  |  |  |  | cDNA | LACK | 6.33858 | 0.01 |
|  |  |  |  |  | cDNA | ATPase | 5.9181 | 0.01 |
|  |  |  |  |  | cDNA | PAT6 | 7.17553 | 0.01 |

**Suppl. Table 2.** RT-PCR analysis showing relative expression w.r.t housekeeping controls, actin (ΔC_T_). NOTE: Separate actin controls were maintained for all 3 gels.

| PAT gene | Sequence | Promastigote specific controls | Sequence |
| --- | --- | --- | --- |
| Hp1_FP | 5’TTCGCCTTCCAGACCAC3’ | LACK_FP | 5'-GCTTCGTGTCGTGTGTGTC-3' |
| Hp1_RP | 5’CAGCCACACTTCCATCG3’ | LACK_RP | 5'-CGTCCTTGGTGTGCTTCA-3' |
| PAT1_FP | 5’CCAACACCACGCAATCA 3’ | ATPase subunit 9_FP | 5′-ATGATGCGCCGTGTCATTTCTCAG-3′ |
| PAT1_RP | 5’GCAGACACAACAGCACGAA3’ | ATPase subunit 9_RP | 5’-ACCGAGAATGGCGTAGTTGAAGAGC-3′ |
| PAT5_FP | 5'AGTGTCGTGGGTTCTCGTC3' | 40S ribosomal subunit_FP | 5′-TGGCTGATTCGAAGAAGGACAAC-3′ |
| PAT5_RP | 5'CCTGCTCTTTGCTGTTTCA3' | 40S ribosomal subunit_RP | 5′-TCGGTTTCCATGAGGGAGAGCAT-3′ |
| PAT7a_FP | 5’ACGACAAGACAGAGCGACA 3’ | Actin_FP | 5’GCCGTCGCTGTACATTGGTA3’ |
| PAT7a_RP | 5’TGGGAAAGTGGGACAGC 3’ | Actin_RP | 5’TGAACGTCATACCCGTCTCC3’ |
| PAT7b_FP | 5'GAGGAAATGTGGTGGAGGT3' |  |  |
| PAT7b_RP | 5'CGGCAATCTGGTAGGAGT3' |  |  |
| PAT6_FP | 5'CATCGCCCTCAACAAACAAC3' |  |  |
| PAT6_RP | 5'CTCAGGCCAAAGAAGGAAAG3' |  |  |
| PAT4_FP | 5'AGTACGTCGTCTTTGGCAAG3' |  |  |
| PAT4_RP | 5'CTGACGGGGATGATGAATAG3' |  |  |
| PAT3_FP | 5'CCTTGTGTTTGTCTCCTTCA3' |  |  |
| PAT3_RP | 5'GCTACCATCGCCTTCTTTC3' |  |  |
| PAT11_FP | 5'GGGATGGCTTGGGTGA3' |  |  |
| PAT11_RP | 5'GAATAGGGCTGTGGTAGGC3' |  |  |
| PAT12_FP | 5'TTCCATACCACCTCTGCTTC3' |  |  |
| PAT12_RP | 5'AAACCATCACCACAACACAG3' |  |  |
| ZnFprot2_FP | 5'GTTCGGGGAGTTGCTGTA3' |  |  |
| ZnFprot2_RP | 5'ATCTGCGGTGTCGTCTGT3' |  |  |
| Hp3_FP | 5'ATACACGCTCGGCTACACC3' |  |  |
| Hp3_RP | 5'ATCTCCACGGCTCTTCTG3' |  |  |
| PAT10_FP | 5'ACCACACCTACCTCATCTCG3' |  |  |
| PAT10_RP | 5'GCAAGTCATCTCCTCCTCAC3' |  |  |
| Hp4_FP | 5'CGACAGCGGAAGCAAT3' |  |  |
| Hp4_RP | 5'GAGAGGGAGAAGAGCGACT3' |  |  |
| PAT2_FP | 5'TCTTCCCCAACACACTCG3' |  |  |
| PAT2_RP | 5'CCAGCCCCACAATCTCA3' |  |  |
| PAT9_FP | 5'TCTTTTGCAGAGAGTGCC3' |  |  |
| PAT9_RP | 5'CGACAGCCAGACAACACC3' |  |  |
| Hp5_FP | 5'TTGCCAAGAATCCTGTGCTT3' |  |  |
| Hp5_RP | 5'AAACCTTGGAGAGAGAGCCT3' |  |  |
| Hp2_FP | 5'AAGGACTACTGCACGACTCT3' |  |  |
| Hp2_RP | 5'AGACGGCGGTACTAAAGA3' |  |  |
| Hp6_FP | 5'TCAAGCCAGCGAACGAG3' |  |  |
| Hp6_RP | 5'CGACAGGAACGACACCA3' |  |  |
| ZnFprot1_FP | 5'GCCATCACAAACTTTGGAGC3' |  |  |
| ZnFprot1_RP | 5'CAGAAGAAGCAGAGCCAGAG3' |  |  |
| FLAM1_FP | 5'AGAAACTCGGTGGATGATGC3' |  |  |
| FLAM1_RP | 5'TTTTGTTCGAGGAGGTGGAC3' |  |  |
| FLAM5_FP | 5'ATCCCTCAATGGCAAACAAG3' |  |  |
| FLAM5_RP | 5'GGTTACGTTTGGGTTGTGCT3' |  |  |
| ODA7_FP | 5'CTGCGGTTTCACCTTGATTC3' |  |  |
| ODA7_RP | 5'ACCGAATGAGGTTGTTCTGC3' |  |  |
| Dynein1_FP | 5'ACGAGCATACAACCCATTCC3' |  |  |
| Dynein1_RP | 5'AGGGAACAACCACGAACAAG3' |  |  |
| Dynein2_FP | 5'ACAGCGGTTTGACAAGTTCC3' |  |  |
| Dynein2_RP | 5'TCCAGAAGGATTCGGTTCAC3' |  |  |
| Dynein4_FP | 5'AAACCCTCGTCGACATCATC3' |  |  |
| Dynein4_RP | 5'TTGAGCACCATCGTCGTTAG3' |  |  |
| Dynein5_FP | 5'TGTGCTGGGAACACTTCATC3' |  |  |
| Dynein5_RP | 5'TCAGGAACCAGTCAATCACG3' |  |  |

**Suppl. Table 3.** Primer sequences of 20 LdPATs, promastigote controls, actin gene and some potentially palmitoylated proteins. FP- Forward primer, RP- Reverse primer

|  | Average Flagella length (μm) | |
| --- | --- | --- |
| **Time (seconds)** | **Control** | **2-BMP** |
| 0.26 | 7.46 | 17.55 |
| 5 | 8.2 | 17.04 |
| 10 | 11.29 | 17.45 |
| 15 | 9.21 | 17.46 |
| 20 | 4.96 | 17.51 |
| 25 | 17.49 | 16.95 |
| 30 | 7.33 | 17.01 |
| 35 | 13.22 | 17.92 |
| 40 | 10.93 | 17.59 |
| 45 | 17.57 | 17.53 |
| 50 | 8.8 | 16.58 |
| 55 | 18.51 | 18.21 |
| 60 | 11.11 | 18.07 |
| Average flagella beat speed (μm/s) | 1.22 | 0.09 |

**Suppl. Table 4.** Average beating speed of flagella (μm/s) calculated of untreated promastigotes and promastigotes treated with 2-BMP.
